# Pre‐Donation Cardiac Arrest and Liver Transplantation Outcomes: Implications for Ischemic Preconditioning

**DOI:** 10.1111/ctr.70309

**Published:** 2025-09-09

**Authors:** Dharesh Raj Amarnath, Samuel J. Tingle, Georgios Kourounis, Chris Freise, Garrett R. Roll, Seiji Yamaguchi, Charles Rickert, Colin H. Wilson

**Affiliations:** ^1^ Newcastle University School of Medicine Newcastle upon Tyne UK; ^2^ Translational and Clinical Research Institute Newcastle University Newcastle upon Tyne UK; ^3^ Institute of Transplantation Freeman Hospital Newcastle upon Tyne UK; ^4^ Department of Surgery, Division of Transplantation University of California San Francisco San Francisco California USA

## Abstract

**Background:**

Liver transplantation is the definitive treatment for end‐stage liver disease and some cancers. The use of livers from donors following pre‐donation cardiac arrest (PDCA), especially with prolonged downtime duration, has been limited outside of the US due to fears over inferior outcomes from ischemic injury. However, PDCA may induce ischemic preconditioning, paradoxically improving post‐transplant outcomes. We analyzed the impact of PDCA occurrence and downtime duration on liver transplantation.

**Methods:**

We used the UNOS registry on adult liver transplantation (2010–2023), and included both donation after brain death (DBD) and donation after circulatory death (DCD) donors. Multivariable regression models were used to analyze the associations. Multiple imputation was used for missing data, and restricted cubic spline modelling to account for non‐linear relationships.

**Results:**

Among 74,592 recipients, 32,631 (43.7%) received a liver from a PDCA donor. PDCA occurrence was associated with a small improvement in graft survival (aHR = 0.914, 95% Cl = 0.851–0.982). Interaction terms revealed this benefit was more pronounced among the following donor groups: DCD, moderately raised alanine aminotransferase (ALT), short admission‐to‐donation time and older donors. These novel associations are all in keeping with a preconditioning effect. Increasing PDCA downtime duration was also associated with a small improvement in graft survival (aHR per doubling = 0.953, 95% Cl = 0.917–0.991). Similar associations were seen with secondary outcomes.

**Conclusions:**

The use of livers from donors with PDCA, including those with prolonged downtime duration, is a safe and simple approach to expand the donor pool internationally. Interaction terms and non‐linear modelling provided clinical evidence for ischemic preconditioning from PDCA, which represents the largest real‐world demonstration of this phenomenon.

## 1 Introduction

Liver transplantation is the definitive treatment for patients with end‐stage liver disease and some cancers [1]. However, liver shortages remain a major challenge, with approximately 12% of patients in the US waiting for more than 5 years for a transplant [2], many of whom either die or are removed from the waitlist. Strategies that expand the deceased‐donor pool or improve organ utilization from existing donors are required to address this issue.

An important approach to improving utilization is by identifying factors that are incorrectly perceived to negatively impact outcomes. One such potential factor is pre‐donation cardiac arrest (PDCA). A substantial proportion of organ donors have histories of cardiac arrest before death. Although livers from many of these donors may meet standard criteria for transplantation, concerns persist that PDCA—specifically if PDCA downtime duration is prolonged—may impact recipient outcomes due to exposure to a period of warm ischemic time, which may result in ischemic liver injury. Conversely, some believe that this ischemia may have a preconditioning effect, which could provide protection against the subsequent ischemic insults that are inevitable during retrieval/transport.

Although the use of livers from PDCA donors is relatively standard practice in the US, they remain underutilized in Europe and the UK. For instance, a single‐center study in the UK found that only 39% of patients following PDCA were even referred for organ donation [3]. Therefore, increasing confidence in the utilization of these livers from PDCA donors in the US setting would support the potential expansion of donor pools outside the US. In this study, we aim to assess the impact of PDCA occurrence and PDCA downtime duration on liver transplant outcomes.

## 2 Methods

This population cohort study was performed using data collected from the United Network for Organ Sharing (UNOS) Registry. Study‐specific ethical review, approval, or informed consent were not required. We included adult (aged 18 years and over at the time of transplant) recipients of liver‐only transplants performed between January 1, 2010, and March 31, 2023. Both donation after brain death (DBD) and donation after circulatory death (DCD) donors were included. Exclusion criteria were multi‐organ transplants, uncontrolled DCD and missing data for PDCA. The primary exposures were PDCA occurrence (binary) and PDCA downtime duration in minutes. Cardiac arrests occurring after planned DCD withdrawal were not assessed in our analysis. Data were extracted on March 31, 2024, which was the common closure date of the study, ensuring all patients had a minimum of 1 year post transplant follow‐up period. Ethnicity was included as a confounder in the multivariable models as it is known to affect post‐transplant outcomes [4].

### 2.1 Outcomes

The primary outcome was recipient 1‐year graft survival, which is defined in the UNOS/STAR registry as graft loss or death. Secondary outcomes were 1‐year patient survival (mortality), graft loss in 30 days (binary), and length of stay (analyzed as time‐to‐discharge). Length of stay was censored at a maximum of 90 days for those still in hospital, or those who had died or lost their graft before 90 days. A sensitivity analysis was performed using graft function at last follow‐up to assess death‐censored graft survival.

### 2.2 Statistical Analysis

To account for missing data, multiple imputation was performed (aregImpute; Hmisc R package) to generate 20 imputed datasets [5]. This uses predictive mean matching with bootstrap draws to build rich additive restricted cubic spline models [6]. This was chosen in preference to multiple imputation by chained equations, as it preserves non‐linear relationships. This was especially important as our outcome models employed non‐linear modelling. For survival outcomes (graft survival, patient survival, and length of stay), we included the event indicator variable and the cumulative hazard of the event in the model, to preserve relationships between outcome and missing covariates [7]. Separate imputed datasets were created for the full cohort and the cohort with PDCA. PDCA occurrence or downtime duration were included as variables in the relevant multiple imputation models, to preserve relationships with potential confounders [8, 9].

To assess the impact of PDCA occurrence (in the full cohort) and PDCA downtime duration (in the cohort of recipients from PDCA donors) on recipient graft survival, patient survival and hospital length of stay, multivariable cox regression was performed. Results were pooled from 20 imputed datasets, adjusting for variance based on both within‐ and between‐imputation variation (using Rubin's rules); fit.mult.impute function (Hmisc package) [10]. Multivariable logistic regression was performed to assess the impact on early graft loss.

Adjustment for a wide range of confounders was performed. Potential confounders were selected based on previous research and clinical expertise; statistical variable selection techniques (e.g., stepwise selection) were avoided [11].

To avoid assumptions of linear associations, restricted cubic splines with four knots (5th, 35th, 65th, and 95th percentiles) were used to analyze continuous variables known to have a strong correlation with the outcome or likely to have non‐linear relationships. An a priori decision was made to use splines for these variables. For other continuous variables, those with significant right skew on visual assessment of histograms were Log2‐transformed (effect estimates therefore relate to a doubling in value).

As the impact of PDCA occurrence and downtime duration on outcome may have differed with certain donor and transplant factors, additional models were built which included interaction terms [12]. Sensitivity analyses were also performed adjusting for additional potential confounders that were not included in the main models due to issues with missing data or multicollinearity [13].

Kaplan–Meier plots were generated to show crude graft and patient survival, stratified by PDCA occurrence. Continuous variables are given as median and interquartile range. Outputs of models are given as effect estimates with 95% confidence intervals. All analyses were performed in R (R Foundation for Statistical Computing, Vienna, Austria) [14], using the following packages; tidyverse, rms, Hmisc, and survminer [5, 6, 15, 16].

## 3 Results

From donors between 2010 and 2023, 74,592 liver recipients were included in the analysis, of whom 32,631 (43.7%) received a liver from a PDCA donor. Information on cohort selection is given in our study flow diagram (Figure S1). Key cohort demographics are given in Table 1. Additional demographics and full description of missing data are in Table S1. We included both DBD (68,701 DBD donors; 92.1% of total cohort) and DCD (5891 DCD donors; 7.9% of total cohort) donors in our study.

**TABLE 1:** Cohort demographic characteristics.

Characteristics	No PDCA donors, No. (%) (*n* = 41,961)	PDCA donors, No. (%) (*n* = 32,631)	Overall, No. (%) (*n* = 74,592)
**Donor age**			
Median [IQR], years	45.0 [30.0, 57.0]	39.0 [28.0, 52.0]	42.0 [29.0, 55.0]
**Donor BMI**			
Median [IQR], kg/m^2^	26.6 [23.2, 30.7]	27.5 [23.8, 32.1]	27.0 [23.5, 31.3]
**Donor sex**			
Female	16002 (38.1%)	13592 (41.7%)	29594 (39.7%)
Male	25959 (61.9%)	19039 (58.3%)	44998 (60.3%)
**Donor ethnicity**			
American Indian/Alaska Native	203 (0.5%)	172 (0.5%)	375 (0.5%)
Asian	1220 (2.9%)	682 (2.1%)	1902 (2.5%)
Black	8131 (19.4%)	5687 (17.4%)	13818 (18.5%)
Hispanic/Latino	6645 (15.8%)	4160 (12.7%)	10805 (14.5%)
Multiracial	117 (0.3%)	102 (0.3%)	219 (0.3%)
Native Hawaiian/other Pacific Islander	124 (0.3%)	75 (0.2%)	199 (0.3%)
White	25521 (60.8%)	21753 (66.7%)	47274 (63.4%)
**Donor cause of death**			
Anoxia	1675 (4.0%)	16447 (50.4%)	18122 (24.3%)
Cerebrovascular/Stroke	20823 (49.6%)	3679 (11.3%)	24502 (32.8%)
Drug overdose	246 (0.6%)	44 (0.1%)	290 (0.4%)
Head trauma	751 (1.8%)	7750 (23.8%)	8501 (11.4%)
CNS tumor	246 (0.6%)	44 (0.1%)	290 (0.4%)
**Donor type**			
DBD	39148 (93.3%)	29553 (90.6%)	68701 (92.1%)
DCD	2813 (6.7%)	3078 (9.4%)	5891 (7.9%)
**Recipient age**			
Median [IQR], years	57.0 [50.0, 63.0]	57.0 [50.0, 63.0]	57.0 [50.0, 63.0]
**Recipient BMI**			
Median [IQR], kg/m^2^	28.1 [24.6, 32.5]	28.2 [24.6, 32.5]	28.2 [24.6, 32.5]
**Recipient sex**			
Female	14453 (34.4%)	10892 (33.4%)	25345 (34.0%)
Male	27508 (65.6%)	21739 (66.6%)	49247 (66.0%)
**Recipient ethnicity**			
American Indian/Alaska Native	345 (0.8%)	260 (0.8%)	605 (0.8%)
Asian	1814 (4.3%)	1359 (4.2%)	3173 (4.3%)
Black	3495 (8.3%)	2696 (8.3%)	6191 (8.3%)
Hispanic/Latino	6560 (15.6%)	4768 (14.6%)	11328 (15.2%)
Multiracial	230 (0.5%)	177 (0.5%)	407 (0.5%)
Native Hawaiian/other Pacific Islander	70 (0.2%)	55 (0.2%)	125 (0.2%)
White	29447 (70.2%)	23316 (71.5%)	52763 (70.7%)
**Recipient primary diagnosis at listing**			
Alcoholic liver disease	11542 (27.5%)	9747 (29.9%)	21289 (28.5%)
HCC	5189 (12.4%)	3998 (12.3%)	9187 (12.3%)
NASH	6037 (14.4%)	4822 (14.8%)	10859 (14.6%)
Cholestatic disease	2979 (7.1%)	2301 (7.1%)	5280 (7.1%)
Acute liver failure	1477 (3.5%)	1028 (3.2%)	2505 (3.4%)
HCV	7301 (17.4%)	5378 (16.5%)	12679 (17.0%)
Other	6148 (14.7%)	4453 (13.6%)	10601 (14.2%)
**Cold ischemic time**			
Median [IQR], min	5.90 [4.65, 7.30]	5.85 [4.70, 7.22]	5.88 [4.67, 7.27]
**Donor ALT values**			
Median [IQR], U/L	37.0 [24.0, 70.0]	159 [75.0, 365]	65.0 [31.0, 178]

Abbreviations: ALT, alanine aminotransferase; BMI, body mass index; DBD, donation after brain death; DCD, donation after circulatory death; HCC, hepatocellular carcinoma; HCV, hepatitis C virus; NASH, nonalcoholic steatohepatitis; PDCA, pre‐donation cardiac arrest.

**FIGURE 1:**
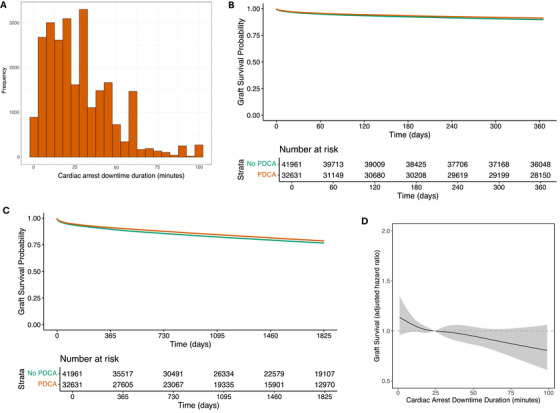
(A) Number of transplants stratified by PDCA downtime duration. (B) Above – Kaplan–Meier curve of 1‐year graft survival stratified by PDCA occurrence. Below – Number at risk over 1 year by PDCA occurrence. (C) Above – Kaplan–Meier curve of 5‐year graft survival stratified by PDCA occurrence. Below – Number at risk over 5 years by PDCA occurrence. (D) Association between 1‐year graft survival plotted against PDCA downtime duration utilizing restricted cubic splines with four knots adjusted for all factors in Table S5.

The median PDCA downtime duration was 24 min (IQR, 12–40 min), with the distribution of downtime duration shown in Figure 1A. Crude 1‐ and 5‐year graft survival data stratified by PDCA occurrence are shown in Kaplan–Meier (Figure 1B,C, respectively).

Donors with PDCA, whose livers were transplanted, tended to have higher peak alanine aminotransferase (ALT) and peak AST values (ALT: median, 160 U/L; IQR, 75–365 U/L and AST: median, 228 U/L; IQR, 111‐508 U/L) than donors without PDCA (ALT: median, 37 U/L; IQR, 24–70 U/L and AST: median, 58, IQR, 36–109 U/L), as shown in Figure S2.

### 3.1 Impact of PDCA Occurrence on 1‐Year Graft Survival

Multivariable cox regression model was used to assess the association of PDCA with recipient 1‐year graft survival, adjusting for a wide range of factors (Table 2). PDCA occurrence was associated with a small, statistically significant, improvement in graft survival (aHR = 0.914, 95% CI = 0.851–0.982, *p* = 0.014). Key continuous potential confounders which were deemed likely to have a non‐linear relationship with graft survival were adjusted for using restricted cubic splines (Figure S3).

**TABLE 2:** Multivariable cox regression model for 1‐year graft survival for the full cohort (*n* = 74,592), pooled from 20 imputed datasets.

Variable	Hazard ratio (95% Cl)	*p* value
**PDCA: Yes**	0.914 (0.851–0.982)	0.014
**Donor age (per 10 years)**	1.070 (1.050–1.090)	<0.001
**Donor sex: Male**	0.996 (0.945–1.049)	0.868
**Donor ethnicity**		
White	Ref	−
Black	1.061 (0.995–1.132)	0.070
Hispanic/Latino	1.027 (0.958–1.101)	0.448
Asian	1.176 (1.025–1.349)	0.021
Other	0.971 (0.761–1.240)	0.815
**Donor BMI (per 5 units)**	1.006 (0.986–1.027)	0.554
**Donor cause of death**		
Anoxia	Ref	−
Cerebrovascular/stroke	1.091 (1.001–1.188)	0.048
CNS tumor	0.835 (0.551–1.265)	0.395
Drug overdose	0.918 (0.835–1.009)	0.076
Head trauma	0.956 (0.875–1.044)	0.315
**Donor type**		
DBD	Ref	−
DCD	1.798 (1.649–1.961)	<0.001
**Donor diabetes status: Present**	1.164 (1.084–1.250)	<0.001
**Donor hypertension: Present**	1.066 (1.004–1.133)	0.037
**Log2‐donor bilirubin**	1.055 (1.003–1.110)	0.037
**Log2‐donor INR**	0.997 (0.940–1.057)	0.923
**Static cold storage solution**		
UW	Ref	−
HTK	1.056 (0.989–1.127)	0.101
Other	0.944 (0.882–1.010)	0.094
**Machine perfusion type**		
None	Ref	−
Normothermic	0.636 (0.480–0.842)	0.002
Hypothermic	0.520 (0.167–1.618)	0.259
Other	0.517 (0.214–1.248)	0.142
**Allocation type**		
Local	Ref	−
Regional	0.977 (0.923–1.033)	0.411
National	1.126 (1.037–1.223)	0.005
**Previous liver TX: Yes**	2.210 (2.021–2.418)	<0.001
**Recipient age (per 10 years)**	1.166 (1.137–1.194)	<0.001
**Recipient sex: Male**	1.041 (0.989–1.096)	0.125
**Recipient ethnicity**		
White	Ref	−
Black	1.303 (1.205–1.410)	<0.001
Hispanic/Latino	0.999 (0.935–1.068)	0.977
Asian, Non‐Hispanic	0.879 (0.775–0.996)	0.043
Other	1.191 (0.992–1.431)	0.061
**Recipient BMI (per 5 units)**	1.024 (1.003–1.046)	0.023
**MELD score (per 10 units)**	1.059 (1.024–1.096)	<0.001
**Status 1A: Yes**	1.037 (0.905–1.188)	0.603
**Recipient primary diagnosis**		
Alcoholic liver disease	Ref	−
HCC	1.189 (1.082–1.308)	<0.001
NASH	1.132 (1.040–1.231)	0.004
Cholestatic disease	1.107 (0.995–1.232)	0.063
Acute liver failure	1.251 (1.079–1.451)	0.003
HCV	1.214 (1.104–1.334)	<0.001
Others	1.310 (1.210–1.419)	<0.001
**Recipient HCV status: Positive**	1.053 (0.977–1.135)	0.176
**Recipient medical condition at TX**		
Not Hospitalized	Ref	−
Hospitalized, but not in ICU	0.989 (0.913–1.071)	0.784
In ICU	1.413 (1.281–1.560)	<0.001
**Recipient functional status at TX** **(per 10 percentage points)** **(10%—Moribund, 100%—Normal)**	0.908 (0.894–0.922)	<0.001
**Pre‐transplant dialysis: Yes**	1.213 (1.121–1.313)	<0.001
**Recipient diabetes**		
No	Ref	−
Type 1	1.149 (0.937–1.408)	0.181
Type 2	1.160 (1.097–1.227)	<0.001
**Log2‐Days on liver waiting list**	1.033 (1.022–1.044)	<0.001
RCS: Donor ALT*	RCS terms	0.018
RCS: Donor creatinine*	RCS terms	0.062
RCS: Year of liver transplantation*	RCS terms	<0.001
RCS: Cold ischemic time*	RCS terms	<0.001
RCS: Donor admission‐to‐retrieval time*	RCS terms	0.012

*Notes:* Right‐skewed variables not modelled with splines were log2‐transformed, so the results relate to the change in 1‐year graft survival every time the variable doubles. * for restricted cubic splines see Figure S3.

Abbreviations: ALT, alanine aminotransferase; BMI, body mass index; DBD, donation after brain death; DCD, donation after circulatory death; HCC: hepatocellular carcinoma; HCV, hepatitis C virus; HTK, histidine‐tryptophan‐ketoglutarate; ICU, intensive care unit; INR, International normalized ratio; MELD, model for end‐stage liver disease; NASH, nonalcoholic steatohepatitis; PDCA, pre‐donation cardiac arrest; TX, transplantation; UW, University of Wisconsin.

Data on macrosteatosis percentage on biopsy was largely missing (*n* = 45,007) as biopsy was not routinely done. Therefore, we excluded it from the main model shown in Table 2. Sensitivity analysis adjusting for macrosteatosis revealed that it was a significant non‐linear predictor of graft survival. Including macrosteatosis did not meaningfully change the effect estimate for the impact of PDCA on graft survival (aHR = 0.917, 95% CI = 0.854–0.986, *p* = 0.019).

To adjust for potential individual transplant center effects, a cox frailty model with random effects for transplant center was performed, revealing significant variation in graft survival by transplant center (*p* < 0.001, Chi^2^ = 234.993, df = 72.357). When adjusting for this between‐center variation, the estimate for the impact of PDCA on graft survival did not meaningfully change (aHR = 0.915, 95% CI = 0.851–0.983, *p* = 0.018). The remainder of the described sensitivity analyses did not change our conclusions.

A model identical to that in Table 2, but instead using graft survival censored at 5 years as the outcome, was consistent with the main results (PDCA aHR = 0.932, 95% CI = 0.886–0.980, *p* = 0.006).

### 3.2 Interaction and Non‐Linear Models Consistent With Ischemic Preconditioning Effect

We hypothesized that the effect of PDCA on graft survival may differ based on other donor or transplant factors. To test this, we added interaction terms to the model in Table 2.

**FIGURE 2:**
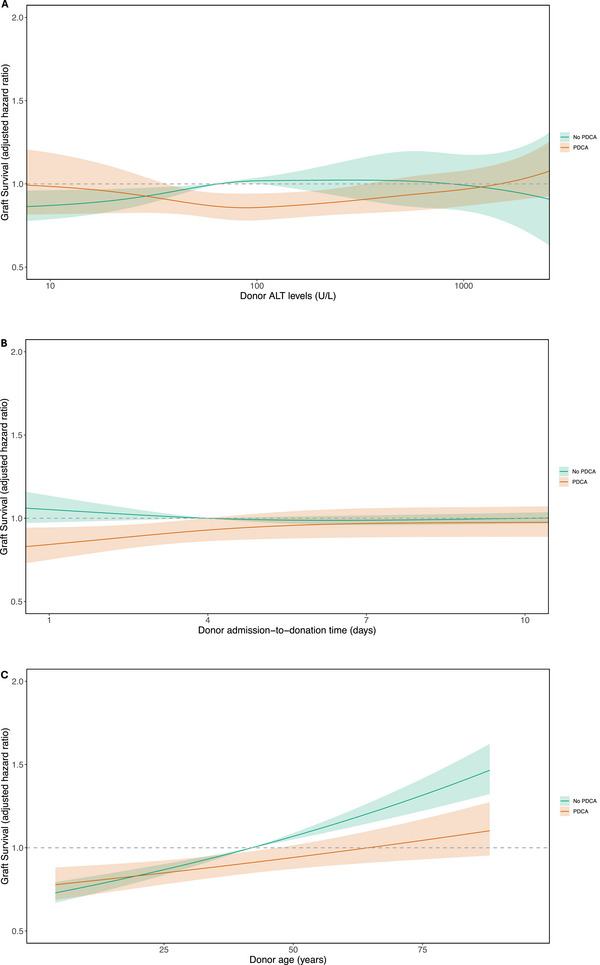
(A) Interaction model between PDCA and donor ALT on 1‐year graft survival. (B) Interaction model between PDCA and time from admission to organ retrieval (donor hospital length of stay) on 1‐year graft survival (C) Interaction model between PDCA and donor age on 1‐year graft survival, all adjusted for factors in Table 2.

The impact of PDCA occurrence was different based on donor peak ALT levels (interaction *p* = 0.044). As shown in Figure 2A, there was no association between PDCA occurrence and graft survival at low donor peak ALT of 40 U/L (aHR = 0.941, 95% CI = 0.865–1.023, *p* = 0.154). But a significant improvement in graft survival was seen at moderately raised ALT of 100 U/L (aHR = 0.842, 95% CI = 0.767–0.925, *p* < 0.001) and 300 U/L (aHR = 0.881, 95% CI = 0.779–0.997, *p* = 0.045). There was again no association in graft survival based on PDCA occurrence at ALT of over 500 U/L. Although ALT was used for its greater liver‐specificity, AST also showed a similar pattern. Overall, this shows that the association between PDCA and improved graft survival is only present when there was biochemical evidence of ischemia.

There was a significant interaction between PDCA occurrence and donor type (interaction *p* = 0.035). A greater benefit was seen in DCD livers (DCD aHR = 0.784, CI = 0.667–0.923, *p* = 0.003) when compared to DBD livers (DBD aHR = 0.926, 95% CI = 0.861–0.996, *p* = 0.039).

The relationship between PDCA occurrence and graft survival was dependent on the time between admission to hospital and organ donation (interaction non‐linear *p* = 0.024). PDCA occurrence showed improved graft survival for donors with a short time from admission to organ donation (aHR for 1 day = 0.801, CI = 0.702–0.913, *p* < 0.001), and this improvement was lost with increasing time. Since PDCA events almost always occur immediately before hospitalization or very shortly after arriving to hospital, we used donor admission time as a surrogate for when the PDCA occurred. We identified a clear temporal relationship in the protective effects of PDCA, and benefits were only seen when donation occurred within about 4 days of hospital admission (as a surrogate for time of PDCA) (Figure 2B).

The impact of PDCA occurrence also differed based on donor age (interaction *p* = 0.010). Improved graft survival was only seen for older donors (aHR for PDCA at age 60 = 0.845, 95% CI = 0.771–0.927, *p* < 0.001), who are known to be more sensitive to ischemic reperfusion injury (Figure 2C). No change in the effect of PDCA was observed based on machine perfusion (interaction *p* = 0.144) or donor cause of death (interaction *p* = 0.334).

### 3.3 Effect of PDCA Occurrence on Secondary Outcomes

The small improvement from PDCA occurrence was also demonstrated for 1‐year patient survival (aHR = 0.922, 95% CI = 0.851–1.000, *p* = 0.049) (Table S2, splines in Figure S4).

A similar magnitude of association was also seen for recipient hospital length of stay (aHR = 1.017, 95% CI = 0.994–1.040, *p* = 0.153) (Table S3, splines in Figure S5) and early graft loss (aOR = 0.900, 95% CI = 0.800–1.012, *p* = 0.078) (Table S4, splines in Figure S6), but these did not reach statistical significance.

Sensitivity analyses were performed to assess the longer‐term impact of PDCA occurrence on outcome. Multivariable cox regression models were built adjusting for the same factors in Table 2, but instead for 2‐year and 5‐year graft and patient survival. PDCA was associated with improved 2‐year and 5‐year graft survival (2‐year graft survival: aHR = 0.902, 95% CI = 0.848–0.960, *p* < 0.001) (5‐year graft survival: aHR = 0.932, 95% CI = 0.886–0.980, *p* = 0.006). Similarly, significant improvement was also seen for 2‐year and 5‐year patient survival (2‐year patient survival: aHR = 0.906, 95% CI = 0.846–0.970, *p* = 0.004) (5‐year patient survival: aHR = 0.933, 95%CI = 0.933–0.985, *p* = 0.012). These findings suggest that benefits seen from PDCA extended to longer‐term outcomes.

### 3.4 Influence of PDCA Downtime Duration on 1‐Year Graft Survival

Following our analysis of PDCA occurrence, we wanted to assess the impact of PDCA downtime duration in the cohort of recipients from PDCA donors. Multivariable cox regression model was used to assess the association of PDCA downtime duration on 1‐year graft survival (model in Table S5, splines in Figure S7). Increasing PDCA downtime duration was associated with a small, statistically significant, improvement in graft survival (aHR per doubling of downtime duration = 0.953, 95% CI = 0.917–0.991, *p* = 0.015).

An additional multivariable cox regression model was performed with PDCA downtime duration modelled using restricted cubic splines, including all variables in Table S5, to assess the nature of any non‐linear trends. Increasing PDCA downtime duration was again associated with a small improvement in graft survival (Figure 1D); there was no cutoff above which inferior graft survival was observed.

Sensitivity analysis adjusting for biopsy macrosteatosis percentage in Table S5 did not impact the effect estimate for PDCA downtime duration (aHR = 0.953, 95% CI = 0.917–0.990, *p* = 0.014).

A model identical to that in Table S5, but instead using graft survival censored at 5 years as the outcome, was again consistent with the main results (aHR per doubling of PDCA downtime duration = 0.964, 95% CI = 0.938–0.990, *p* = 0.008).

### 3.5 PDCA Downtime Duration and Secondary Outcomes

Increasing PDCA downtime duration was associated with a small improvement in patient survival (aHR per doubling of duration = 0.954, 95% CI = 0.913–0.996, *p* = 0.032) (Table S6, splines in Figure S8). A separate model performed with PDCA downtime duration as a spline did not reveal any cutoff beyond which increasing PDCA downtime duration was associated with worse patient survival (Figure S9A).

Models for hospital length of stay (Table S7, splines in Figure S10) and early graft loss (Table S8, splines in Figure S11) revealed no association between PDCA downtime duration and either of these outcomes. Again, increasing PDCA downtime duration modelled as a spline was not associated with prolonged length of stay (Figure S9B) or early graft loss (Figure S9C).

## 4 Discussion

We have found that PDCA occurrence and increasing downtime duration are associated with small improvement in liver graft survival on multivariable regressions adjusting for a wide range of factors. Therefore, it is safe to transplant livers from donors with PDCA, even when downtime is prolonged within reasonable limits and donor transaminases are raised. These conclusions are held with restricted cubic spline modelling (for non‐linear associations), interaction terms and sensitivity analyses. We have also identified nuanced patterns in the data that provide insightful clinical evidence for liver ischemic preconditioning.

Whilst organs from PDCA donors are frequently utilized in the US, this is not the case in many other parts of the world. For example, in the UK, only 39% of patients who experienced PDCA were referred for organ donation, with only 24% of those referred went on to donate at least one organ [3]. Given that PDCA liver transplants in the US demonstrated favorable results, this could help promote wider acceptance of PDCA donors globally. There is a clear opportunity for other countries to learn from the US experience and expand their deceased donor pool by increasing the utilization of PDCA donors. Over in Europe, where a higher proportion of donors are older and DCD, our finding that the benefit from PDCA was greatest in these donor groups highlights the relevance of this study to the European setting, despite differences from the US donor cohort.

There are two possible explanations for the association of PDCA with improved outcomes. One is residual selection bias despite adjusted analyses, which is possible if important confounders were missing from the multivariable models. However, any selection bias would also apply to other known donor risk factors such as donor age, donor type (DCD vs. DBD), donor diabetes, donor hypertension, CIT, and so forth. All these factors have remained associated with significantly worse graft survival in our models while PDCA is uniquely associated with improved graft survival, making residual selection bias an unlikely explanation for the beneficial effect.

An alternative explanation is ischemic preconditioning; a phenomenon in which short periods of non‐lethal ischemia have been shown to protect the organ during a subsequent sustained ischemia [17]. The beneficial effects of ischemic preconditioning include decrease in liver necrosis, anti‐apoptotic effects, preservation of liver microcirculation and improvement in graft survival [18]. Therefore, the hepatic ischemia during PDCA may provide hepatocellular protection against subsequent ischemia associated with the retrieval, cold preservation, and transportation of clinical liver transplantation.

Our models with interaction terms are also consistent with the concept of ischemic preconditioning. We identified a dose‐response relationship between donor peak ALT levels and the impact of PDCA on improved graft survival (Figure 2A). At low donor ALT (absence of acute hepatic ischemic injury), there is no association between PDCA and graft survival. However, at moderately raised donor ALT, there is a significant association between PDCA and improved graft survival. And there is a loss of effect at ALT of over 500 U/L. This dose‐response relationship aligns with the theory of ischemic preconditioning in PDCA, where a certain level of ischemia (hepatic ischemic injury) is required to trigger protective mechanisms, but excessive ischemic damage may overwhelm these pathways. The loss of effect at very high ALT suggests that selection bias is unlikely to explain this relationship; if selection bias were the primary driver, the spline in Figure 2A would continue to diverge with increasing donor peak ALT.

The association between PDCA and improved outcomes was found to be dependent on the time between initial admission and organ donation (Figure 2B). The protective benefits of PDCA were only seen when the time from admission to organ donation was short. Since most PDCA events occur before hospitalization or early during the hospital stay, this time‐sensitive relationship suggests that preconditioning benefits are lost if organ donation does not occur shortly after the PDCA. No effect is observed if donation occurred a week after PDCA (Figure 2B).

There was also significant interaction between PDCA and donor type. The association between PDCA and improved graft survival was far greater in DCD livers, which also have far greater ischemia as part of the retrieval process. It could also be more aggressive selection bias in the DCD setting but given extensive adjustment of potential confounders in our models, this pattern is less likely to be solely due to selection bias. In our study, DCD donors make up a small proportion of the total cohort, accounting for only 7.9% (5891 DCD donors) of the cohort.

The protective effects of PDCA were more pronounced in livers from older donors (Figure 2C). Organs from older donors are typically more susceptible to ischemia‐reperfusion injury (IRI) [19], and this suggests that PDCA may be beneficial in improving graft survival by reducing the detrimental effects of IRI.

The PDCA downtime duration spline modelling (Figures 1A and S9) further revealed that the association with improved outcome increased with a continuous increase in the downtime duration. Again, this is consistent with ischemia from PDCA having a preconditioning effect.

Some existing research on the impact of PDCA have shown potential non‐significant trends toward improved liver graft survival [20, 21, 22]. However, these results have been mixed or inconclusive. In contrast, we demonstrate a robust and statistically significant association between PDCA and improved outcomes—findings that have not been shown previously. Moreover, these studies had sample sizes of at least 40‐fold lower than our study, making it impossible to test whether this association is independent with any certainty.

We used restricted cubic spline modelling to capture non‐linear relationships without splitting continuous variables into arbitrary categories, thereby avoiding the limitations of predefined categories in previous studies. Additionally, we uniquely included interaction terms in our analyses to explore how the effects of PDCA occurrence and downtime duration varied with certain factors such as donor ALT, donor age, donor type, and time of donor PDCA. These advanced modelling approaches have demonstrated nuanced associations which support ischemic preconditioning.

Machine perfusion is increasingly changing the landscape of liver transplantation in the United States [23]. Therefore, we adjusted for the use of machine perfusion as a confounder in our analysis. The ability of machine to assess organ viability will likely give additional confidence in accepting livers from PDCA donors with prolonged downtime duration.

The main limitation is the inevitable degree of missing data, but robust techniques were employed to impute this data. As discussed, selection bias is an inherent concern. Although we adjusted for a wide range of factors, there may be important additional confounders which are not adequately reported in the UNOS data. Information on how return of spontaneous circulation was achieved is not available. The precise duration of PDCA downtime is difficult to verify, as it is typically estimated—a representation of real‐world clinical practice.

## 5 Conclusion

This study is the largest analysis of the impact of PDCA on liver transplantation. Our findings show that recipients of livers from donors with PDCA, even with prolonged downtime duration, do not experience inferior post‐transplant outcomes. Given the relatively high prevalence of PDCA, and reluctance to use livers from these donors outside of the US, accepting and utilizing more livers from PDCA donors could increase the donor pool, ultimately saving more lives.

If an ischemic preconditioning effect were present, we would expect the benefit to be greatest in DCD donors (who experience the most ischemia), in those with moderately raised ALT following PDCA (biochemical evidence of liver ischemia), and in those that proceed to donation shortly after the PDCA. We show these exact nuanced patterns in the data by combining non‐linear modelling and interaction analyses. Overall, this represents the largest real‐world demonstration of ischemic preconditioning.

## Author Contributions

Samuel J. Tingle had full access to all the data in the study and takes responsibility for the integrity of the data and the accuracy of the data analysis. Concept and design: S.T. Acquisition and cleaning of data: S.T., D.A. Statistical analysis: S.T., D.A. Interpretation of data: All authors. Drafting of the manuscript: D.A. Critical review of the manuscript for important intellectual content: All authors. Supervision: S.T., C.W.

## Conflicts of Interest

The authors declare no conflicts of interest.

## Supporting information




**Supporting File 1:** ctr70309‐sup‐0001‐SuppMat.docx

## Data Availability

The data used in the analysis are available from OPTN/UNOS. Data can be requested from OPTN/UNOS through the following website: https://optn.transplant.hrsa.gov/data/view‐data‐reports/request‐data/.
