# Supplementary material for: Pre‐Donation Cardiac Arrest and Liver Transplantation Outcomes: Implications for Ischemic Preconditioning
Source: Clin Transplant. 2025 Sep 9;39(9):e70309. doi: 10.1111/ctr.70309 (PMC12419763; doi:10.1111/ctr.70309)
Supplement: Supplementary file 1 — Supporting File 1: ctr70309‐sup‐0001‐SuppMat.docx [file CTR-39-e70309-s001.docx]

# Supplementary material

Supplementary Table 1: Extended cohort demographic characteristics. Abbreviations: PDCA, pre-donation cardiac arrest; BMI, body mass index; UW, University of Wisconsin; HTK, histidine-tryptophan-ketoglutarate; DBD, donation after brain death; DCD, donation after circulatory death; HBV, hepatitis B virus; CMV, cytomegalovirus; HIV, human immunodeficiency virus; ICU, Intensive care unit; MELD, model for end-stage liver disease; HCC, hepatocellular carcinoma; NASH, nonalcoholic steatohepatitis; HCV, hepatitis C virus; ALT, alanine aminotransferase; INR, international normalized ratio; PTT, partial thromboplastin time.

|  | No PDCA donors, No. (%) (N=41,961) | PDCA donors, No. (%) (N=32,631) | Overall, No. (%) (N=74,592) |
| --- | --- | --- | --- |
| **Donor Age** |  |  |  |
| Median [IQR], y | 45.0 [30.0, 57.0] | 39.0 [28.0, 52.0] | 42.0 [29.0, 55.0] |
| **Donor BMI** |  |  |  |
| Median [IQR], kg/m2 | 26.6 [23.2, 30.7] | 27.5 [23.8, 32.1] | 27.0 [23.5, 31.3] |
| Missing | 318 (0.8%) | 453 (1.4%) | 771 (1.0%) |
| **Donor admission-to-retrieval time** |  |  |  |
| Median [IQR], days | 4.00 [3.00, 6.00] | 4.00 [3.00, 6.00] | 4.00 [3.00, 6.00] |
| Missing | 134 (0.3%) | 102 (0.3%) | 236 (0.3%) |
| **Donor Sex** |  |  |  |
| Female | 16002 (38.1%) | 13592 (41.7%) | 29594 (39.7%) |
| Male | 25959 (61.9%) | 19039 (58.3%) | 44998 (60.3%) |
| **Cause of Donor Death** |  |  |  |
| Anoxia | 1675 (4.0%) | 16447 (50.4%) | 18122 (24.3%) |
| Cerebrovascular/stroke | 20823 (49.6%) | 3679 (11.3%) | 24502 (32.8%) |
| CNS Tumor | 246 (0.6%) | 44 (0.1%) | 290 (0.4%) |
| Drug overdose | 751 (1.8%) | 7750 (23.8%) | 8501 (11.4%) |
| Head Trauma | 17531 (41.8%) | 4296 (13.2%) | 21827 (29.3%) |
| Missing | 935 (2.2%) | 415 (1.3%) | 1350 (1.8%) |
| **Static Cold Storage Solution** |  |  |  |
| UW | 28231 (67.3%) | 21581 (66.1%) | 49812 (66.8%) |
| HTK | 6319 (15.1%) | 5337 (16.4%) | 11656 (15.6%) |
| Other | 7411 (17.7%) | 5713 (17.5%) | 13124 (17.6%) |
| **Donor type** |  |  |  |
| DBD | 39148 (93.3%) | 29553 (90.6%) | 68701 (92.1%) |
| DCD | 2813 (6.7%) | 3078 (9.4%) | 5891 (7.9%) |
| **MELD Lab Score at Transplant** |  |  |  |
| Median [IQR] | 23.0 [14.0, 32.0] | 23.0 [14.0, 32.0] | 23.0 [14.0, 32.0] |
| Missing | 9 (0.0%) | 8 (0.0%) | 17 (0.0%) |
| **Recipient Serum Albumin at Transplant** |  |  |  |
| Median [IQR], g/dL | 3.10 [2.60, 3.60] | 3.10 [2.70, 3.60] | 3.10 [2.70, 3.60] |
| Missing | 47 (0.1%) | 27 (0.1%) | 74 (0.1%) |
| **Recipient Encephalopathy at Transplant** |  |  |  |
| None | 15434 (36.8%) | 11840 (36.3%) | 27274 (36.6%) |
| Grade 1-2 | 21117 (50.3%) | 16581 (50.8%) | 37698 (50.5%) |
| Grade 3-4 | 5405 (12.9%) | 4207 (12.9%) | 9612 (12.9%) |
| Missing | 5 (0.0%) | 3 (0.0%) | 8 (0.0%) |
| **Recipient Ascites at Transplant** |  |  |  |
| Absent | 10684 (25.5%) | 8164 (25.0%) | 18848 (25.3%) |
| Slight | 17913 (42.7%) | 13987 (42.9%) | 31900 (42.8%) |
| Moderate | 13359 (31.8%) | 10477 (32.1%) | 23836 (32.0%) |
| Missing | 5 (0.0%) | 3 (0.0%) | 8 (0.0%) |
| **Recipient Pre-Transplant Dialysis** |  |  |  |
| No | 36694 (87.4%) | 28629 (87.7%) | 65323 (87.6%) |
| Yes | 5164 (12.3%) | 3869 (11.9%) | 9033 (12.1%) |
| Missing | 103 (0.2%) | 133 (0.4%) | 236 (0.3%) |
| **Recipient Age** |  |  |  |
| Median [IQR], y | 57.0 [50.0, 63.0] | 57.0 [50.0, 63.0] | 57.0 [50.0, 63.0] |
| **Recipient BMI** |  |  |  |
| Median [IQR], kg/m2 | 28.1 [24.6, 32.5] | 28.2 [24.6, 32.5] | 28.2 [24.6, 32.5] |
| Missing | 126 (0.3%) | 84 (0.3%) | 210 (0.3%) |
| **Recipient Sex** |  |  |  |
| Female | 14453 (34.4%) | 10892 (33.4%) | 25345 (34.0%) |
| Male | 27508 (65.6%) | 21739 (66.6%) | 49247 (66.0%) |
| **Recipient HCV Status** |  |  |  |
| Negative | 29174 (69.5%) | 23003 (70.5%) | 52177 (69.9%) |
| Positive | 11903 (28.4%) | 9069 (27.8%) | 20972 (28.1%) |
| Missing | 884 (2.1%) | 559 (1.7%) | 1443 (1.9%) |
| **Recipient HBV Status** |  |  |  |
| Negative | 33747 (80.4%) | 26719 (81.9%) | 60466 (81.1%) |
| Positive | 6792 (16.2%) | 5055 (15.5%) | 11847 (15.9%) |
| Missing | 1422 (3.4%) | 857 (2.6%) | 2279 (3.1%) |
| **Recipient HBV Surface Antigen** |  |  |  |
| Negative | 39259 (93.6%) | 30862 (94.6%) | 70121 (94.0%) |
| Positive | 1752 (4.2%) | 1213 (3.7%) | 2965 (4.0%) |
| Missing | 950 (2.3%) | 556 (1.7%) | 1506 (2.0%) |
| **Treated for Rejection within 1 Year** |  |  |  |
| No | 28141 (67.1%) | 22548 (69.1%) | 50689 (68.0%) |
| Yes | 3652 (8.7%) | 2715 (8.3%) | 6367 (8.5%) |
| Missing | 10168 (24.2%) | 7368 (22.6%) | 17536 (23.5%) |
| **Treated for Rejection within 6 Months** |  |  |  |
| No | 30086 (71.7%) | 24108 (73.9%) | 54194 (72.7%) |
| Yes | 3158 (7.5%) | 2339 (7.2%) | 5497 (7.4%) |
| Missing | 8717 (20.8%) | 6184 (19.0%) | 14901 (20.0%) |
| **Recipient CMV Status at Transplant** |  |  |  |
| Negative | 14448 (34.4%) | 12026 (36.9%) | 26474 (35.5%) |
| Positive | 26579 (63.3%) | 19933 (61.1%) | 46512 (62.4%) |
| Missing | 934 (2.2%) | 672 (2.1%) | 1606 (2.2%) |
| **Recipient HIV Serostatus** |  |  |  |
| Negative | 40178 (95.8%) | 31367 (96.1%) | 71545 (95.9%) |
| Positive | 238 (0.6%) | 232 (0.7%) | 470 (0.6%) |
| Missing | 1545 (3.7%) | 1032 (3.2%) | 2577 (3.5%) |
| **Recipient Medical Condition at Listing** |  |  |  |
| Not Hospitalized | 26630 (63.5%) | 21061 (64.5%) | 47691 (63.9%) |
| Hospitalized, but not in ICU | 8791 (21.0%) | 6873 (21.1%) | 15664 (21.0%) |
| In ICU | 6539 (15.6%) | 4697 (14.4%) | 11236 (15.1%) |
| Missing | 1 (0.0%) | 0 (0%) | 1 (0.0%) |
| **Allocation Type** |  |  |  |
| Local | 25640 (61.1%) | 18940 (58.0%) | 44580 (59.8%) |
| Regional | 11697 (27.9%) | 9075 (27.8%) | 20772 (27.8%) |
| National | 4624 (11.0%) | 4616 (14.1%) | 9240 (12.4%) |
| **Donor History of Diabetes** |  |  |  |
| No | 36365 (86.7%) | 27896 (85.5%) | 64261 (86.2%) |
| Yes | 5231 (12.5%) | 4425 (13.6%) | 9656 (12.9%) |
| Missing | 365 (0.9%) | 310 (1.0%) | 675 (0.9%) |
| **Recipient History of Diabetes** |  |  |  |
| No | 30527 (72.8%) | 23773 (72.9%) | 54300 (72.8%) |
| Type 1 | 467 (1.1%) | 354 (1.1%) | 821 (1.1%) |
| Type 2 | 10173 (24.2%) | 7933 (24.3%) | 18106 (24.3%) |
| Missing | 794 (1.9%) | 571 (1.7%) | 1365 (1.8%) |
| **Donor History of Hypertension** |  |  |  |
| No | 24390 (58.1%) | 21847 (67.0%) | 46237 (62.0%) |
| Yes | 17171 (40.9%) | 10452 (32.0%) | 27623 (37.0%) |
| Missing | 400 (1.0%) | 332 (1.0%) | 732 (1.0%) |
| **Donor Peak Serum Sodium Values** |  |  |  |
| Median [IQR], mEq/L | 157 [150, 164] | 155 [148, 161] | 156 [149, 163] |
| Missing | 60 (0.1%) | 64 (0.2%) | 124 (0.2%) |
| **Liver Machine Perfusion Type** |  |  |  |
| No machine perfusion | 41491 (98.9%) | 32139 (98.5%) | 73630 (98.7%) |
| Normothermic | 389 (0.9%) | 418 (1.3%) | 807 (1.1%) |
| Hypothermic | 28 (0.1%) | 31 (0.1%) | 59 (0.1%) |
| Unspecified | 53 (0.1%) | 43 (0.1%) | 96 (0.1%) |
| **Super-Urgent Candidate Status at Transplant** |  |  |  |
| NotStatus1a | 40515 (96.6%) | 31611 (96.9%) | 72126 (96.7%) |
| Status1a | 1446 (3.4%) | 1020 (3.1%) | 2466 (3.3%) |
| **Recipient Primary Diagnosis at Listing** |  |  |  |
| Alcoholic Liver Disease | 11542 (27.5%) | 9747 (29.9%) | 21289 (28.5%) |
| HCC | 5189 (12.4%) | 3998 (12.3%) | 9187 (12.3%) |
| NASH | 6037 (14.4%) | 4822 (14.8%) | 10859 (14.6%) |
| Cholestatic Disease | 2979 (7.1%) | 2301 (7.1%) | 5280 (7.1%) |
| Acute Liver Failure | 1477 (3.5%) | 1028 (3.2%) | 2505 (3.4%) |
| HCV | 7301 (17.4%) | 5378 (16.5%) | 12679 (17.0%) |
| Others/Unknown | 6148 (14.7%) | 4453 (13.6%) | 10601 (14.2%) |
| Missing | 1288 (3.1%) | 904 (2.8%) | 2192 (2.9%) |
| **Graft Loss within 30 Days** |  |  |  |
| No | 40252 (95.9%) | 31518 (96.6%) | 71770 (96.2%) |
| Yes | 1671 (4.0%) | 1088 (3.3%) | 2759 (3.7%) |
| Missing | 38 (0.1%) | 25 (0.1%) | 63 (0.1%) |
| **Recipient Functional Status Percentage at Transplant** |  |  |  |
| Median [IQR], % | 50.0 [30.0, 70.0] | 50.0 [30.0, 70.0] | 50.0 [30.0, 70.0] |
| Missing | 382 (0.9%) | 395 (1.2%) | 777 (1.0%) |
| **Previous Liver Transplants** |  |  |  |
| No | 40149 (95.7%) | 31237 (95.7%) | 71386 (95.7%) |
| Yes | 1812 (4.3%) | 1394 (4.3%) | 3206 (4.3%) |
| **Donor Ethnicity** |  |  |  |
| American Indian/Alaska Native | 203 (0.5%) | 172 (0.5%) | 375 (0.5%) |
| Asian | 1220 (2.9%) | 682 (2.1%) | 1902 (2.5%) |
| Black | 8131 (19.4%) | 5687 (17.4%) | 13818 (18.5%) |
| Hispanic/Latino | 6645 (15.8%) | 4160 (12.7%) | 10805 (14.5%) |
| Multiracial | 117 (0.3%) | 102 (0.3%) | 219 (0.3%) |
| Native Hawaiian/other Pacific Islander | 124 (0.3%) | 75 (0.2%) |  |
| White | 25521 (60.8%) | 21753 (66.7%) | 47274 (63.4%) |
| **Recipient Ethnicity** |  |  |  |
| American Indian/Alaska Native | 345 (0.8%) | 260 (0.8%) | 605 (0.8%) |
| Asian | 1814 (4.3%) | 1359 (4.2%) | 3173 (4.3%) |
| Black | 3495 (8.3%) | 2696 (8.3%) | 6191 (8.3%) |
| Hispanic/Latino | 6560 (15.6%) | 4768 (14.6%) | 11328 (15.2%) |
| Multiracial | 230 (0.5%) | 177 (0.5%) | 407 (0.5%) |
| Native Hawaiian/other Pacific Islander | 70 (0.2%) | 55 (0.2%) | 125 (0.2%) |
| White | 29447 (70.2%) | 23316 (71.5%) | 52763 (70.7%) |
| **Liver Waitlist Duration** |  |  |  |
| Median [IQR], days | 77.0 [11.0, 266] | 73.0 [11.0, 261] | 75.0 [11.0, 264] |
| Missing | 140 (0.3%) | 88 (0.3%) | 228 (0.3%) |
| **Deceased Donor Macro Fat Percentage** |  |  |  |
| Median [IQR], % | 5.00 [0, 10.0] | 5.00 [0, 10.0] | 5.00 [0, 10.0] |
| Missing | 26027 (62.0%) | 18980 (58.2%) | 45007 (60.3%) |
| **Deceased Donor Micro Fat Percentage** |  |  |  |
| Median [IQR], % | 5.00 [0, 10.0] | 5.00 [0, 10.0] | 5.00 [0, 10.0] |
| Missing | 26729 (63.7%) | 19515 (59.8%) | 46244 (62.0%) |
| **Total Cold Ischemic Time** |  |  |  |
| Median [IQR], mins | 5.90 [4.65, 7.30] | 5.85 [4.70, 7.22] | 5.88 [4.67, 7.27] |
| Missing | 211 (0.5%) | 160 (0.5%) | 371 (0.5%) |
| **Donor Peak AST values** |  |  |  |
| Median [IQR], U/L | 58.0 [36.0, 108] | 228 [111, 506] | 99.0 [48.0, 255] |
| Missing | 16 (0.0%) | 47 (0.1%) | 63 (0.1%) |
| **Donor Peak ALT values** |  |  |  |
| Median [IQR], U/L | 37.0 [24.0, 70.0] | 159 [75.0, 365] | 65.0 [31.0, 178] |
| Missing | 8 (0.0%) | 13 (0.0%) | 21 (0.0%) |
| **Donor Peak Serum Creatinine values** |  |  |  |
| Median [IQR], mg/dL | 1.33 [1.00, 1.93] | 1.70 [1.30, 2.90] | 1.50 [1.10, 2.30] |
| Missing | 0 (0%) | 1 (0.0%) | 1 (0.0%) |
| **Donor Peak Bilirubin values** |  |  |  |
| Median [IQR], mg/dL | 1.00 [0.700, 1.60] | 0.900 [0.600, 1.40] | 1.00 [0.700, 1.50] |
| Missing | 42 (0.1%) | 37 (0.1%) | 79 (0.1%) |
| **Donor Peak Conjugated Bilirubin values** |  |  |  |
| Median [IQR], mg/dL | 0.300 [0.200, 0.500] | 0.300 [0.200, 0.500] | 0.300 [0.200, 0.500] |
| Missing | 1893 (4.5%) | 1233 (3.8%) | 3126 (4.2%) |
| **Donor Peak Prothrombin Time** |  |  |  |
| Median [IQR], secs | 16.3 [14.0, 19.0] | 16.5 [14.2, 19.2] | 16.4 [14.1, 19.1] |
| Missing | 398 (0.9%) | 258 (0.8%) | 656 (0.9%) |
| **Donor Peak INR values** |  |  |  |
| Median [IQR] | 1.40 [1.20, 1.62] | 1.40 [1.22, 1.70] | 1.40 [1.20, 1.68] |
| Missing | 153 (0.4%) | 138 (0.4%) | 291 (0.4%) |
| **Donor Peak PTT values** |  |  |  |
| Median [IQR], secs | 34.8 [30.0, 41.5] | 37.7 [32.0, 47.3] | 36.0 [31.0, 44.0] |
| Missing | 722 (1.7%) | 908 (2.8%) | 1630 (2.2%) |
| **Donor inotropic support** |  |  |  |
| No | 22,469 (53.5%) | 18,164 (55.7%) | 40,633 (54.5%) |
| Yes | 19,427 (46.3%) | 14,402 (44.1%) | 33,829 (45.3%) |
| Missing | 65 (0.2%) | 65 (0.2%) | 130 (0.2%) |

Supplementary Table 2: Multivariable cox regression model for 1-year patient survival for the full cohort (n=74592), pooled from 20 imputed datasets (n=74592). Right-skewed variables not modelled with splines were log2-transformed, so the results relate to the change in 1-year patient survival every time the variable doubles. * for restricted cubic splines see Supplementary Figure 4. PDCA = Pre-donation cardiac arrest; BMI = Body mass index; DBD = Donation after brain death; DCD = Donation after circulatory death; INR = International normalized ratio; UW = University of Wisconsin; HTK = histidine-tryptophan-ketoglutarate; TX = Transplantation; MELD = Model for end-stage liver disease; HCC: Hepatocellular carcinoma; NASH = Nonalcoholic steatohepatitis; HCV = Hepatitis C virus; ICU = Intensive care unit; ALT = Alanine aminotransferase.

| Variable | Hazard ratio (95% Cl) | P value |
| --- | --- | --- |
| **PDCA: yes** | 0.922 (0.851 to 1.000) | 0.049 |
| **Donor age (per 10 years)** | 1.060 (1.038 to 1.082) | <0.001 |
| **Donor sex: male** | 1.036 (0.977 to 1.099) | 0.235 |
| **Donor ethnicity** |  |  |
| White | Ref | - |
| Black | 1.018 (0.946 to 1.095) | 0.633 |
| Hispanic/Latino | 0.997 (0.923 to 1.078) | 0.949 |
| Asian | 1.072 (0.911 to 1.260) | 0.402 |
| Other | 0.927 (0.700 to 1.227) | 0.595 |
| **Donor BMI (per 5 units)** | 1.024 (1.000 to 1.048) | 0.047 |
| **Donor cause of death** |  |  |
| Anoxia | Ref | - |
| Cerebrovascular/stroke | 1.034 (0.939 to 1.139) | 0.492 |
| CNS Tumor | 0.695 (0.423 to 1.143) | 0.152 |
| Drug Overdose | 0.970 (0.875 to 1.075) | 0.564 |
| Head Trauma | 0.903 (0.817 to 0.998) | 0.045 |
| **Donor type** |  |  |
| DBD | Ref | - |
| DCD | 1.390 (1.249 to 1.547) | <0.001 |
| **Donor diabetes status: present** | 1.123 (1.035 to 1.219) | 0.006 |
| **Donor hypertension: present** | 1.016 (0.949 to 1.088) | 0.653 |
| **Log2-Donor bilirubin** | 1.055 (0.996 to 1.117) | 0.066 |
| **Log2-Donor INR** | 0.992 (0.929 to 1.060) | 0.815 |
| **Static cold storage solution** |  |  |
| UW | Ref | - |
| HTK | 1.068 (0.993 to 1.149) | 0.075 |
| Other | 0.946 (0.876 to 1.021) | 0.154 |
| **Machine perfusion type** |  |  |
| None | Ref | - |
| Normothermic | 0.627 (0.448 to 0.877) | 0.006 |
| Hypothermic | 0.758 (0.243 to 2.360) | 0.632 |
| Other | 0.599 (0.224 to 1.605) | 0.309 |
| **Allocation type** |  |  |
| Local | Ref | - |
| Regional | 0.959 (0.901 to 1.022) | 0.197 |
| National | 1.113 (1.014 to 1.223) | 0.025 |
| **Previous liver TX: yes** | 2.461 (2.234 to 2.712) | <0.001 |
| **Recipient age (per 10 years)** | 1.286 (1.250 to 1.323) | <0.001 |
| **Recipient sex: male** | 0.998 (0.943 to 1.057) | 0.958 |
| **Recipient ethnicity** |  |  |
| White | Ref | - |
| Black | 1.303 (1.193 to 1.423) | <0.001 |
| Hispanic/Latino | 0.964 (0.894 to 1.040) | 0.344 |
| Asian | 0.827 (0.716 to 0.956) | 0.010 |
| Other | 1.218 (0.992 to 1.495) | 0.060 |
| **Recipient BMI (per 5 units)** | 0.998 (0.975 to 1.022) | 0.873 |
| **MELD score (per 10 units)** | 1.097 (1.057 to 1.139) | <0.001 |
| **Status 1A: yes** | 1.001 (0.864 to 1.160) | 0.989 |
| **Recipient primary diagnosis** |  |  |
| Alcoholic Liver Disease | Ref | - |
| HCC | 1.236 (1.110 to 1.375) | <0.001 |
| NASH | 1.171 (1.065 to 1.288) | 0.001 |
| Cholestatic Disease | 0.931 (0.817 to 1.061) | 0.284 |
| Acute Liver Failure | 1.310 (1.115 to 1.540) | 0.001 |
| HCV | 1.238 (1.115 to 1.376) | <0.001 |
| Others | 1.310 (1.196 to 1.434) | <0.001 |
| **Recipient HCV status: positive** | 1.103 (1.015 to 1.199) | 0.021 |
| **Recipient medical condition at TX** |  |  |
| Not Hospitalized | Ref | - |
| Hospitalized, but not in ICU | 1.012 (0.926 to 1.107) | 0.788 |
| In ICU | 1.393 (1.248 to 1.555) | <0.001 |
| **Recipient functional status at TX**  **(per 10 percentage points)**  **(10% - Moribund, 100% - Normal)** | 0.886 (0.870 to 0.902) | <0.001 |
| **Pre-transplant dialysis: yes** | 1.245 (1.143 to 1.356) | <0.001 |
| **Recipient diabetes** |  |  |
| No | Ref | - |
| Type 1 | 1.335 (1.078 to 1.655) | 0.008 |
| Type 2 | 1.211 (1.138 to 1.288) | <0.001 |
| **Log2-Days on liver waiting list** | 1.029 (1.017 to 1.042) | <0.001 |
| **RCS: Donor ALT*** | RCS terms | 0.028 |
| **RCS: Donor creatinine*** | RCS terms | 0.171 |
| **RCS: Year of liver transplantation*** | RCS terms | <0.001 |
| **RCS: Cold ischemic time*** | RCS terms | <0.001 |
| **RCS: Donor admission-to-retrieval time*** | RCS terms | 0.032 |

Supplementary Table 3: Multivariable cox regression model for length of stay for the full cohort, pooled from 20 imputed datasets (n=74592). Right-skewed variables not modelled with splines were log2-transformed, so the results relate to the change in length of stay every time the variable doubles. * for restricted cubic splines see Supplementary Figure 5. PDCA = Pre-donation cardiac arrest; BMI = Body mass index; DBD = Donation after brain death; DCD = Donation after circulatory death; INR = International normalized ratio; UW = University of Wisconsin; HTK = histidine-tryptophan-ketoglutarate; TX = Transplantation; MELD = Model for end-stage liver disease; HCC: Hepatocellular carcinoma; NASH = Nonalcoholic steatohepatitis; HCV = Hepatitis C virus; ICU = Intensive care unit; ALT = Alanine aminotransferase.

| Variable | Hazard ratio (95% Cl) | P value |
| --- | --- | --- |
| **PDCA: yes** | 1.017 (0.994 to 1.040) | 0.153 |
| **Donor age (per 10 years)** | 0.987 (0.981 to 0.993) | <0.001 |
| **Donor sex: male** | 1.015 (0.998 to 1.032) | 0.078 |
| **Donor ethnicity** |  |  |
| White | Ref | - |
| Black | 0.981 (0.961 to 1.001) | 0.066 |
| Hispanic/Latino | 0.975 (0.953 to 0.998) | 0.031 |
| Asian | 0.929 (0.884 to 0.975) | 0.003 |
| Other | 1.026 (0.954 to 1.104) | 0.489 |
| **Donor BMI (per 5 units)** | 0.998 (0.991 to 1.005) | 0.534 |
| **Donor cause of death** |  |  |
| Anoxia | Ref | - |
| Cerebrovascular/stroke | 0.925 (0.899 to 0.951) | <0.001 |
| CNS Tumor | 0.984 (0.869 to 1.113) | 0.793 |
| Drug Overdose | 1.007 (0.980 to 1.035) | 0.614 |
| Head Trauma | 0.988 (0.961 to 1.016) | 0.410 |
| **Donor type** |  |  |
| DBD | Ref | - |
| DCD | 0.915 (0.888 to 0.943) | <0.001 |
| **Donor diabetes status: present** | 0.922 (0.899 to 0.945) | <0.001 |
| **Donor hypertension: present** | 0.996 (0.976 to 1.016) | 0.679 |
| **Log2-Donor bilirubin** | 0.975 (0.959 to 0.991) | 0.002 |
| **Log2-Donor INR** | 0.990 (0.972 to 1.009) | 0.321 |
| **Static cold storage solution** |  |  |
| UW | Ref | - |
| HTK | 0.943 (0.923 to 0.963) | <0.001 |
| Other | 1.066 (1.044 to 1.088) | <0.001 |
| **Machine perfusion type** |  |  |
| None | Ref | - |
| Normothermic | 1.238 (1.146 to 1.336) | <0.001 |
| Hypothermic | 1.476 (1.137 to 1.915) | 0.003 |
| Other | 1.342 (1.095 to 1.645) | 0.005 |
| **Allocation type** |  |  |
| Local | Ref | - |
| Regional | 1.029 (1.010 to 1.048) | 0.002 |
| National | 0.923 (0.899 to 0.947) | <0.001 |
| **Previous liver TX: yes** | 0.669 (0.642 to 0.697) | <0.001 |
| **Recipient age (per 10 years)** | 0.942 (0.935 to 0.949) | <0.001 |
| **Recipient sex: male** | 1.056 (1.039 to 1.074) | <0.001 |
| **Recipient ethnicity** |  |  |
| White | Ref | - |
| Black | 0.882 (0.858 to 0.908) | <0.001 |
| Hispanic/Latino | 0.952 (0.932 to 0.973) | <0.001 |
| Asian | 0.925 (0.890 to 0.962) | <0.001 |
| Other | 0.976 (0.918 to 1.038) | 0.436 |
| **Recipient BMI (per 5 units)** | 0.981 (0.975 to 0.988) | <0.001 |
| **MELD score (per 10 units)** | 0.880 (0.870 to 0.890) | <0.001 |
| **Status 1A: yes** | 0.977 (0.923 to 1.034) | 0.426 |
| **Recipient primary diagnosis** |  |  |
| Alcoholic Liver Disease | Ref | - |
| HCC | 1.130 (1.098 to 1.163) | <0.001 |
| NASH | 0.971 (0.945 to 0.996) | 0.025 |
| Cholestatic Disease | 1.020 (0.987 to 1.053) | 0.245 |
| Acute Liver Failure | 1.008 (0.956 to 1.062) | 0.777 |
| HCV | 1.021 (0.990 to 1.053) | 0.181 |
| Others | 0.953 (0.929 to 0.978) | <0.001 |
| **Recipient HCV status: positive** | 0.987 (0.963 to 1.011) | 0.288 |
| **Recipient medical condition at TX** |  |  |
| Not Hospitalized | Ref | - |
| Hospitalized, but not in ICU | 0.842 (0.821 to 0.862) | <0.001 |
| In ICU | 0.662 (0.640 to 0.685) | <0.001 |
| **Recipient functional status at TX**  **(per 10 percentage points)**  **(10% - Moribund, 100% - Normal)** | 1.063 (1.058 to 1.068) | <0.001 |
| **Pre-transplant dialysis: yes** | 0.828 (0.804 to 0.853) | <0.001 |
| **Recipient diabetes** |  |  |
| No | Ref | - |
| Type 1 | 0.889 (0.826 to 0.956) | 0.001 |
| Type 2 | 0.968 (0.950 to 0.987) | <0.001 |
| **Log2-Days on liver waiting list** | 0.971 (0.968 to 0.975) | <0.001 |
| **RCS: Donor ALT*** | RCS terms | <0.001 |
| **RCS: Donor creatinine*** | RCS terms | 0.098 |
| **RCS: Year of liver transplantation*** | RCS terms | <0.001 |
| **RCS: Cold ischemic time*** | RCS terms | <0.001 |
| **RCS: Donor admission-to-retrieval time*** | RCS terms | <0.001 |

Supplementary Table 4: Multivariable logistic linear regression model for graft loss in 30 days for the full cohort, pooled from 20 imputed datasets (n=74592). Right-skewed variables not modelled with splines were log2-transformed, so the results relate to the change in graft loss in 30 days every time the variable doubles. * for restricted cubic splines see Supplementary Figure 6. PDCA = Pre-donation cardiac arrest; BMI = Body mass index; DBD = Donation after brain death; DCD = Donation after circulatory death; INR = International normalized ratio; UW = University of Wisconsin; HTK = histidine-tryptophan-ketoglutarate; TX = Transplantation; MELD = Model for end-stage liver disease; HCC: Hepatocellular carcinoma; NASH = Nonalcoholic steatohepatitis; HCV = Hepatitis C virus; ICU = Intensive care unit; ALT = Alanine aminotransferase.

| Variable | Odds ratio (95% Cl) | P value |
| --- | --- | --- |
| **PDCA: yes** | 0.900 (0.800 to 1.012) | 0.078 |
| **Donor age (per 10 years)** | 1.022 (0.991 to 1.055) | 0.167 |
| **Donor sex: male** | 0.957 (0.878 to 1.043) | 0.320 |
| **Donor ethnicity** |  |  |
| White | Ref | - |
| Black | 1.038 (0.933 to 1.156) | 0.491 |
| Hispanic/Latino | 1.007 (0.898 to 1.128) | 0.910 |
| Asian | 1.312 (1.054 to 1.634) | 0.015 |
| Other | 0.800 (0.524 to 1.221) | 0.300 |
| **Donor BMI (per 5 units)** | 1.078 (1.043 to 1.115) | <0.001 |
| **Donor cause of death** |  |  |
| Anoxia | Ref | - |
| Cerebrovascular/stroke | 1.263 (1.096 to 1.456) | 0.001 |
| CNS Tumor | 1.179 (0.650 to 2.139) | 0.588 |
| Drug Overdose | 0.895 (0.765 to 1.047) | 0.166 |
| Head Trauma | 1.043 (0.901 to 1.208) | 0.571 |
| **Donor type** |  |  |
| DBD | Ref | - |
| DCD | 1.672 (1.440 to 1.940) | <0.001 |
| **Donor diabetes status: present** | 1.191 (1.056 to 1.344) | 0.004 |
| **Donor hypertension: present** | 1.026 (0.927 to 1.136) | 0.619 |
| **Log2-Donor bilirubin** | 1.031 (0.948 to 1.121) | 0.479 |
| **Log2-Donor INR** | 0.997 (0.905 to 1.099) | 0.959 |
| **Static cold storage solution** |  |  |
| UW | Ref | - |
| HTK | 1.214 (1.092 to 1.350) | <0.001 |
| Other | 0.978 (0.874 to 1.093) | 0.691 |
| **Machine perfusion type** |  |  |
| None | Ref | - |
| Normothermic | 0.533 (0.332 to 0.857) | 0.009 |
| Hypothermic or Other | 0.151 (0.021 to 1.086) | 0.060 |
| **Allocation type** |  |  |
| Local | Ref | - |
| Regional | 0.924 (0.841 to 1.014) | 0.097 |
| National | 1.220 (1.068 to 1.395) | 0.003 |
| **Previous liver TX: yes** | 2.785 (2.425 to 3.199) | <0.001 |
| **Recipient age (per 10 years)** | 1.096 (1.054 to 1.139) | <0.001 |
| **Recipient sex: male** | 0.997 (0.916 to 1.085) | 0.948 |
| **Recipient ethnicity** |  |  |
| White | Ref | - |
| Black | 1.348 (1.184 to 1.535) | <0.001 |
| Hispanic/Latino | 1.068 (0.957 to 1.191) | 0.238 |
| Asian | 1.114 (0.920 to 1.350) | 0.269 |
| Other | 1.030 (0.748 to 1.419) | 0.857 |
| **Recipient BMI (per 5 units)** | 1.157 (1.120 to 1.196) | <0.001 |
| **MELD score (per 10 units)** | 1.006 (0.952 to 1.064) | 0.820 |
| **Status 1A: yes** | 1.525 (1.253 to 1.857) | <0.001 |
| **Recipient primary diagnosis** |  |  |
| Alcoholic Liver Disease | Ref | - |
| HCC | 1.195 (1.019 to 1.402) | 0.029 |
| NASH | 1.118 (0.969 to 1.289) | 0.126 |
| Cholestatic Disease | 1.252 (1.053 to 1.489) | 0.011 |
| Acute Liver Failure | 1.439 (1.157 to 1.789) | 0.001 |
| HCV | 1.131 (0.956 to 1.337) | 0.151 |
| Others | 1.458 (1.280 to 1.659) | <0.001 |
| **Recipient HCV status: positive** | 0.947 (0.833 to 1.076) | 0.404 |
| **Recipient medical condition at TX** |  |  |
| Not Hospitalized | Ref | - |
| Hospitalized, but not in ICU | 0.921 (0.804 to 1.054) | 0.230 |
| In ICU | 1.478 (1.256 to 1.739) | <0.001 |
| **Recipient functional status at TX**  **(per 10 percentage points)**  **(10% - Moribund, 100% - Normal)** | 0.989 (0.986 to 0.991) | <0.001 |
| **Pre-transplant dialysis: yes** | 1.279 (1.125 to 1.455) | <0.001 |
| **Recipient diabetes** |  |  |
| No | Ref | - |
| Type 1 | 0.968 (0.679 to 1.382) | 0.860 |
| Type 2 | 1.002 (0.911 to 1.102) | 0.966 |
| **Log2-Days on liver waiting list** | 1.060 (1.041 to 1.079) | <0.001 |
| **RCS: Donor ALT*** | RCS terms | 0.003 |
| **RCS: Donor creatinine*** | RCS terms | 0.176 |
| **RCS: Year of liver transplantation*** | RCS terms | <0.001 |
| **RCS: Cold ischemic time*** | RCS terms | <0.001 |
| **RCS: Donor admission-to-retrieval time*** | RCS terms | 0.089 |

Supplementary Table 5: Multivariable cox regression model for 1-year graft survival to assess impact of PDCA downtime duration (PDCA cohort; n=32631), pooled from 20 imputed datasets. right-skewed variables not modelled with splines were log2-transformed, so the results relate to the change in 1-year graft loss every time the variable doubles. * For restricted cubic splines see Supplementary Figure 7. PDCA = Pre-donation cardiac arrest; BMI = Body mass index; DBD = Donation after brain death; DCD = Donation after circulatory death; INR = International normalized ratio; UW = University of Wisconsin; HTK = Histidine-tryptophan-ketoglutarate; TX = Transplantation; MELD = Model for end-stage liver disease; HCC: Hepatocellular carcinoma; NASH = Nonalcoholic steatohepatitis; HCV = Hepatitis C virus; ICU = Intensive care unit; ALT = Alanine aminotransferase.

| Variable | Hazard ratio (95% Cl) | P value |
| --- | --- | --- |
| **Log2-PDCA downtime duration** | 0.953 (0.917 to 0.991) | 0.015 |
| **Donor age (per 10 years)** | 1.053 (1.021 to 1.086) | <0.001 |
| **Donor sex: male** | 1.021 (0.942 to 1.107) | 0.606 |
| **Donor ethnicity** |  |  |
| White | Ref | - |
| Black | 1.113 (1.005 to 1.233) | 0.041 |
| Hispanic/Latino | 1.061 (0.948 to 1.188) | 0.304 |
| Asian | 1.207 (0.950 to 1.533) | 0.124 |
| Other | 1.179 (0.834 to 1.667) | 0.352 |
| **Donor BMI (per 5 units)** | 1.022 (0.990 to 1.056) | 0.171 |
| **Donor cause of death** |  |  |
| Anoxia | Ref | - |
| Cerebrovascular/stroke | 1.024 (0.898 to 1.167) | 0.725 |
| CNS Tumor | 0.486 (0.121 to 1.945) | 0.308 |
| Drug Overdose | 0.912 (0.823 to 1.010) | 0.076 |
| Head Trauma | 0.950 (0.830 to 1.088) | 0.458 |
| **Donor type** |  |  |
| DBD | Ref | - |
| DCD | 1.655 (1.453 to 1.884) | <0.001 |
| **Donor diabetes status: present** | 1.181 (1.053 to 1.324) | 0.004 |
| **Donor hypertension: present** | 1.021 (0.926 to 1.126) | 0.671 |
| **Log2-Donor bilirubin** | 1.056 (0.972 to 1.146) | 0.196 |
| **Log2-Donor INR** | 0.998 (0.909 to 1.096) | 0.965 |
| **Static cold storage solution** |  |  |
| UW | Ref | - |
| HTK | 1.062 (0.959 to 1.176) | 0.246 |
| Other | 0.959 (0.861 to 1.068) | 0.447 |
| **Machine perfusion type** |  |  |
| None | Ref | - |
| Normothermic | 0.530 (0.337 to 0.834) | 0.006 |
| Hypothermic | 0.341 (0.048 to 2.407) | 0.280 |
| Other | 1.005 (0.373 to 2.709) | 0.992 |
| **Allocation type** |  |  |
| Local | Ref | - |
| Regional | 0.947 (0.866 to 1.036) | 0.236 |
| National | 1.193 (1.057 to 1.346) | 0.004 |
| **Previous liver TX: yes** | 2.388 (2.079 to 2.744) | <0.001 |
| **Recipient age (per 10 years)** | 1.172 (1.127 to 1.218) | <0.001 |
| **Recipient sex: male** | 0.978 (0.902 to 1.060) | 0.589 |
| **Recipient ethnicity** |  |  |
| White | Ref | - |
| Black | 1.417 (1.254 to 1.600) | <0.001 |
| Hispanic/Latino | 1.003 (0.902 to 1.116) | 0.955 |
| Asian | 1.024 (0.845 to 1.240) | 0.810 |
| Other | 1.366 (1.040 to 1.793) | 0.025 |
| **Recipient BMI (per 5 units)** | 1.038 (1.005 to 1.072) | 0.025 |
| **MELD score (per 10 units)** | 1.061 (1.005 to 1.120) | 0.031 |
| **Status 1A: yes** | 0.946 (0.763 to 1.172) | 0.610 |
| **Recipient primary diagnosis** |  |  |
| Alcoholic Liver Disease | Ref | - |
| HCC | 1.290 (1.112 to 1.498) | <0.001 |
| NASH | 1.169 (1.024 to 1.335) | 0.021 |
| Cholestatic Disease | 1.059 (0.893 to 1.257) | 0.509 |
| Acute Liver Failure | 1.335 (1.063 to 1.676) | 0.013 |
| HCV | 1.353 (1.163 to 1.572) | <0.001 |
| Others | 1.337 (1.177 to 1.518) | <0.001 |
| **Recipient HCV status: positive** | 1.026 (0.910 to 1.157) | 0.674 |
| **Recipient medical condition at TX** |  |  |
| Not Hospitalized | Ref | - |
| Hospitalized, but not in ICU | 1.083 (0.957 to 1.227) | 0.208 |
| In ICU | 1.626 (1.394 to 1.896) | <0.001 |
| **Recipient functional status at TX**  **(per 10 percentage points)**  **(10% - Moribund, 100% - Normal)** | 0.915 (0.892 to 0.937) | <0.001 |
| **Pre-transplant dialysis: yes** | 1.248 (1.102 to 1.413) | <0.001 |
| **Recipient diabetes** |  |  |
| No | Ref | - |
| Type 1 | 1.240 (0.905 to 1.700) | 0.181 |
| Type 2 | 1.201 (1.100 to 1.311) | <0.001 |
| **Log2-Days on liver waiting list** | 1.044 (1.026 to 1.062) | <0.001 |
| **RCS: Donor ALT*** | RCS terms | 0.028 |
| **RCS: Donor creatinine*** | RCS terms | 0.571 |
| **RCS: Year of liver transplantation*** | RCS terms | <0.001 |
| **RCS: Cold ischemic time*** | RCS terms | <0.001 |
| **RCS: Donor** **admission-to-retrieval time*** | RCS terms | 0.405 |

Supplementary Table 6: Multivariable cox regression model for 1-year patient survival for the PDCA cohort, pooled from 20 imputed datasets (n=32631). Right-skewed variables not modelled with splines were log2-transformed, so the results relate to the change in 1-year patient loss every time the variable doubles. * for restricted cubic splines see Supplementary Figure 8. PDCA = Pre-donation cardiac arrest; BMI = Body mass index; DBD = Donation after brain death; DCD = Donation after circulatory death; INR = International normalized ratio; UW = University of Wisconsin; HTK = histidine-tryptophan-ketoglutarate; TX = Transplantation; MELD = Model for end-stage liver disease; HCC: Hepatocellular carcinoma; NASH = Nonalcoholic steatohepatitis; HCV = Hepatitis C virus; ICU = Intensive care unit; ALT = Alanine aminotransferase.

| Variable | Hazard ratio (95% Cl) | P value |
| --- | --- | --- |
| **Log2-PDCA downtime duration** | 0.954 (0.913 to 0.996) | 0.032 |
| **Donor age (per 10 years)** | 1.053 (1.017 to 1.089) | 0.003 |
| **Donor sex: male** | 1.051 (0.961 to 1.150) | 0.274 |
| **Donor ethnicity** |  |  |
| White | Ref | - |
| Black | 1.062 (0.946 to 1.192) | 0.310 |
| Hispanic/Latino | 1.024 (0.902 to 1.163) | 0.710 |
| Asian | 1.119 (0.849 to 1.474) | 0.425 |
| Other | 1.332 (0.926 to 1.917) | 0.122 |
| **Donor BMI (per 5 units)** | 1.035 (0.999 to 1.073) | 0.054 |
| **Donor cause of death** |  |  |
| Anoxia | Ref | - |
| Cerebrovascular/stroke | 1.005 (0.869 to 1.163) | 0.943 |
| CNS Tumor | 0.289 (0.041 to 2.048) | 0.214 |
| Drug Overdose | 0.975 (0.872 to 1.090) | 0.655 |
| Head Trauma | 0.857 (0.735 to 1.001) | 0.051 |
| **Donor type** |  |  |
| DBD | Ref | - |
| DCD | 1.288 (1.100 to 1.508) | 0.002 |
| **Donor diabetes status: present** | 1.085 (0.952 to 1.236) | 0.221 |
| **Donor hypertension: present** | 0.985 (0.884 to 1.098) | 0.787 |
| **Log2-Donor bilirubin** | 1.060 (0.968 to 1.162) | 0.209 |
| **Log2-Donor INR** | 0.954 (0.857 to 1.063) | 0.396 |
| **Static cold storage solution** |  |  |
| UW | Ref | - |
| HTK | 1.089 (0.973 to 1.219) | 0.140 |
| Other | 0.964 (0.855 to 1.087) | 0.551 |
| **Machine perfusion type** |  |  |
| None | Ref | - |
| Normothermic | 0.590 (0.357 to 0.976) | 0.040 |
| Hypothermic | 0.480 (0.067 to 3.436) | 0.465 |
| Other | 1.466 (0.543 to 3.958) | 0.450 |
| **Allocation type** |  |  |
| Local | Ref | - |
| Regional | 0.931 (0.843 to 1.029) | 0.162 |
| National | 1.183 (1.033 to 1.354) | 0.015 |
| **Previous liver TX: yes** | 2.687 (2.313 to 3.121) | <0.001 |
| **Recipient age (per 10 years)** | 1.291 (1.234 to 1.350) | <0.001 |
| **Recipient sex: male** | 0.935 (0.855 to 1.022) | 0.141 |
| **Recipient ethnicity** |  |  |
| White | Ref | - |
| Black | 1.424 (1.243 to 1.631) | <0.001 |
| Hispanic/Latino | 0.969 (0.861 to 1.092) | 0.610 |
| Asian | 0.973 (0.782 to 1.211) | 0.807 |
| Other | 1.428 (1.060 to 1.925) | 0.019 |
| **Recipient BMI (per 5 units)** | 1.012 (0.976 to 1.050) | 0.512 |
| **MELD score (per 10 units)** | 1.114 (1.049 to 1.182) | <0.001 |
| **Status 1A: yes** | 0.891 (0.707 to 1.123) | 0.328 |
| **Recipient primary diagnosis** |  |  |
| Alcoholic Liver Disease | Ref | - |
| HCC | 1.355 (1.147 to 1.601) | <0.001 |
| NASH | 1.248 (1.079 to 1.444) | 0.003 |
| Cholestatic Disease | 0.886 (0.723 to 1.088) | 0.248 |
| Acute Liver Failure | 1.426 (1.113 to 1.827) | 0.005 |
| HCV | 1.387 (1.175 to 1.638) | <0.001 |
| Others | 1.327 (1.150 to 1.531) | <0.001 |
| **Recipient HCV status: positive** | 1.080 (0.946 to 1.232) | 0.256 |
| **Recipient medical condition at TX** |  |  |
| Not Hospitalized | Ref | - |
| Hospitalized, but not in ICU | 1.080 (0.941 to 1.240) | 0.273 |
| In ICU | 1.584 (1.337 to 1.877) | <0.001 |
| **Recipient functional status at TX**  **(per 10 percentage points)**  **(10% - Moribund, 100% - Normal)** | 0.898 (0.873 to 0.923) | <0.001 |
| **Pre-transplant dialysis: yes** | 1.264 (1.105 to 1.445) | <0.001 |
| **Recipient diabetes** |  |  |
| No | Ref | - |
| Type 1 | 1.389 (0.991 to 1.947) | 0.057 |
| Type 2 | 1.237 (1.124 to 1.362) | <0.001 |
| **Log2-Days on liver waiting list** | 1.039 (1.020 to 1.059) | <0.001 |
| **RCS: Donor ALT*** | RCS terms | 0.013 |
| **RCS: Donor creatinine*** | RCS terms | 0.668 |
| **RCS: Year of liver transplantation*** | RCS terms | <0.001 |
| **RCS: Cold ischemic time*** | RCS terms | 0.001 |
| **RCS: Donor admission-to-retrieval time*** | RCS terms | 0.882 |

Supplementary Table 7: Multivariable cox regression model for length of stay for the PDCA cohort, pooled from 20 imputed datasets (n=32631). Right-skewed variables not modelled with splines were log2-transformed, so the results relate to the change in length of stay every time the variable doubles. * for restricted cubic splines see Supplementary Figure 10. PDCA = Pre-donation cardiac arrest; BMI = Body mass index; DBD = Donation after brain death; DCD = Donation after circulatory death; INR = International normalized ratio; UW = University of Wisconsin; HTK = histidine-tryptophan-ketoglutarate; TX = Transplantation; MELD = Model for end-stage liver disease; HCC: Hepatocellular carcinoma; NASH = Nonalcoholic steatohepatitis; HCV = Hepatitis C virus; ICU = Intensive care unit; ALT = Alanine aminotransferase.

| Variable | Hazard ratio (95% Cl) | P value |
| --- | --- | --- |
| **Log2-PDCA downtime duration** | 1.005 (0.993 to 1.018) | 0.413 |
| **Donor age (per 10 years)** | 0.987 (0.978 to 0.996) | 0.007 |
| **Donor sex: male** | 1.004 (0.979 to 1.029) | 0.775 |
| **Donor ethnicity** |  |  |
| White | Ref | - |
| Black | 0.974 (0.944 to 1.006) | 0.105 |
| Hispanic/Latino | 0.968 (0.934 to 1.004) | 0.078 |
| Asian | 0.910 (0.840 to 0.987) | 0.022 |
| Other | 0.980 (0.877 to 1.095) | 0.726 |
| **Donor BMI (per 5 units)** | 0.990 (0.980 to 1.000) | 0.044 |
| **Donor cause of death** |  |  |
| Anoxia | Ref | - |
| Cerebrovascular/stroke | 0.937 (0.898 to 0.977) | 0.002 |
| CNS Tumor | 1.253 (0.919 to 1.709) | 0.154 |
| Drug Overdose | 1.002 (0.973 to 1.032) | 0.893 |
| Head Trauma | 0.983 (0.943 to 1.025) | 0.432 |
| **Donor type** |  |  |
| DBD | Ref | - |
| DCD | 0.920 (0.883 to 0.959) | <0.001 |
| **Donor diabetes status: present** | 0.942 (0.908 to 0.978) | 0.002 |
| **Donor hypertension: present** | 1.001 (0.972 to 1.032) | 0.932 |
| **Log2-Donor bilirubin** | 0.966 (0.942 to 0.991) | 0.009 |
| **Log2-Donor INR** | 0.993 (0.965 to 1.023) | 0.662 |
| **Static cold storage solution** |  |  |
| UW | Ref | - |
| HTK | 0.960 (0.931 to 0.991) | 0.013 |
| Other | 1.063 (1.030 to 1.097) | <0.001 |
| **Machine perfusion type** |  |  |
| None | Ref | - |
| Normothermic | 1.271 (1.141 to 1.416) | <0.001 |
| Hypothermic | 1.499 (1.046 to 2.148) | 0.028 |
| Other | 1.191 (0.874 to 1.622) | 0.269 |
| **Allocation type** |  |  |
| Local | Ref | - |
| Regional | 1.043 (1.014 to 1.072) | 0.003 |
| National | 0.922 (0.888 to 0.957) | <0.001 |
| **Previous liver TX: yes** | 0.653 (0.613 to 0.695) | <0.001 |
| **Recipient age (per 10 years)** | 0.937 (0.927 to 0.948) | <0.001 |
| **Recipient sex: male** | 1.085 (1.059 to 1.113) | <0.001 |
| **Recipient ethnicity** |  |  |
| White | Ref | - |
| Black | 0.839 (0.804 to 0.876) | <0.001 |
| Hispanic/Latino | 0.956 (0.925 to 0.989) | 0.008 |
| Asian | 0.896 (0.845 to 0.950) | <0.001 |
| Other | 0.977 (0.890 to 1.072) | 0.621 |
| **Recipient BMI (per 5 units)** | 0.980 (0.970 to 0.989) | <0.001 |
| **MELD score (per 10 units)** | 0.878 (0.863 to 0.892) | <0.001 |
| **Status 1A: yes** | 0.987 (0.903 to 1.078) | 0.766 |
| **Recipient primary diagnosis** |  |  |
| Alcoholic Liver Disease | Ref | - |
| HCC | 1.098 (1.051 to 1.147) | <0.001 |
| NASH | 0.965 (0.928 to 1.004) | 0.077 |
| Cholestatic Disease | 1.012 (0.964 to 1.063) | 0.626 |
| Acute Liver Failure | 1.011 (0.931 to 1.098) | 0.798 |
| HCV | 0.987 (0.943 to 1.033) | 0.573 |
| Others | 0.952 (0.915 to 0.991) | 0.016 |
| **Recipient HCV status: positive** | 0.998 (0.962 to 1.035) | 0.910 |
| **Recipient medical condition at TX** |  |  |
| Not Hospitalized | Ref | - |
| Hospitalized, but not in ICU | 0.833 (0.803 to 0.864) | <0.001 |
| In ICU | 0.651 (0.618 to 0.685) | <0.001 |
| **Recipient functional status at TX**  **(per 10 percentage points)**  **(10% - Moribund, 100% - Normal)** | 1.064 (1.057 to 1.072) | <0.001 |
| **Pre-transplant dialysis: yes** | 0.813 (0.777 to 0.850) | <0.001 |
| **Recipient diabetes** |  |  |
| No | Ref | - |
| Type 1 | 0.843 (0.755 to 0.942) | 0.003 |
| Type 2 | 0.958 (0.930 to 0.986) | 0.004 |
| **Log2-Days on liver waiting list** | 0.972 (0.967 to 0.977) | <0.001 |
| **RCS: Donor ALT*** | RCS terms | 0.255 |
| **RCS: Donor creatinine*** | RCS terms | 0.283 |
| **RCS: Year of liver transplantation*** | RCS terms | <0.001 |
| **RCS: Cold ischemic time*** | RCS terms | <0.001 |
| **RCS: Donor admission-to-retrieval time*** | RCS terms | <0.001 |

Supplementary Table 8: Multivariable logistic linear regression model for graft loss in 30 days for the PDCA cohort, pooled from 20 imputed datasets (n=32631). Right-skewed variables not modelled with splines were log2-transformed, so the results relate to the change in graft loss in 30 days every time the variable doubles. * for restricted cubic splines see Supplementary Figure 11. PDCA = Pre-donation cardiac arrest; BMI = Body mass index; DBD = Donation after brain death; DCD = Donation after circulatory death; INR = International normalized ratio; UW = University of Wisconsin; HTK = histidine-tryptophan-ketoglutarate; TX = Transplantation; MELD = Model for end-stage liver disease; HCC: Hepatocellular carcinoma; NASH = Nonalcoholic steatohepatitis; HCV = Hepatitis C virus; ICU = Intensive care unit; ALT = Alanine aminotransferase.

| Variable | Odds ratio (95% Cl) | P value |
| --- | --- | --- |
| **Log2-PDCA downtime duration** | 0.957 (0.899 to 1.018) | 0.165 |
| **Donor age (per 10 years)** | 1.012 (0.961 to 1.066) | 0.646 |
| **Donor sex: male** | 0.969 (0.847 to 1.109) | 0.649 |
| **Donor ethnicity** |  |  |
| White | Ref | - |
| Black | 1.105 (0.931 to 1.311) | 0.255 |
| Hispanic/Latino | 1.076 (0.894 to 1.296) | 0.438 |
| Asian | 1.320 (0.900 to 1.936) | 0.155 |
| Other | 0.926 (0.499 to 1.716) | 0.806 |
| **Donor BMI (per 5 units)** | 1.090 (1.034 to 1.149) | 0.001 |
| **Donor cause of death** |  |  |
| Anoxia | Ref | - |
| Cerebrovascular/stroke | 1.171 (0.943 to 1.454) | 0.153 |
| CNS Tumor | 0.649 (0.088 to 4.793) | 0.672 |
| Drug Overdose | 0.908 (0.765 to 1.078) | 0.269 |
| Head Trauma | 1.059 (0.849 to 1.322) | 0.609 |
| **Donor type** |  |  |
| DBD | Ref | - |
| DCD | 1.746 (1.404 to 2.170) | <0.001 |
| **Donor diabetes status: present** | 1.252 (1.032 to 1.518) | 0.023 |
| **Donor hypertension: present** | 0.948 (0.803 to 1.120) | 0.529 |
| **Log2-Donor bilirubin** | 1.017 (0.885 to 1.168) | 0.813 |
| **Log2-Donor INR** | 1.008 (0.861 to 1.180) | 0.921 |
| **Static cold storage solution** |  |  |
| UW | Ref | - |
| HTK | 1.136 (0.958 to 1.347) | 0.142 |
| Other | 1.008 (0.844 to 1.204) | 0.928 |
| **Machine perfusion type** |  |  |
| None | Ref | - |
| Yes | 0.377 (0.179 to 0.795) | 0.010 |
| **Allocation type** |  |  |
| Local | Ref | - |
| Regional | 0.871 (0.749 to 1.014) | 0.075 |
| National | 1.329 (1.091 to 1.619) | 0.005 |
| **Previous liver TX: yes** | 2.803 (2.248 to 3.494) | <0.001 |
| **Recipient age (per 10 years)** | 1.089 (1.024 to 1.158) | 0.006 |
| **Recipient sex: male** | 0.914 (0.799 to 1.045) | 0.188 |
| **Recipient ethnicity** |  |  |
| White | Ref | - |
| Black | 1.509 (1.231 to 1.849) | <0.001 |
| Hispanic/Latino | 1.097 (0.922 to 1.306) | 0.297 |
| Asian | 1.396 (1.045 to 1.865) | 0.024 |
| Other | 1.191 (0.739 to 1.918) | 0.473 |
| **Recipient BMI (per 5 units)** | 1.184 (1.124 to 1.248) | <0.001 |
| **MELD score (per 10 units)** | 0.990 (0.905 to 1.084) | 0.830 |
| **Status 1A: yes** | 1.211 (0.876 to 1.675) | 0.246 |
| **Recipient primary diagnosis** |  |  |
| Alcoholic Liver Disease | Ref | - |
| HCC | 1.286 (0.995 to 1.663) | 0.055 |
| NASH | 1.136 (0.910 to 1.417) | 0.260 |
| Cholestatic Disease | 1.257 (0.958 to 1.649) | 0.099 |
| Acute Liver Failure | 1.614 (1.138 to 2.289) | 0.007 |
| HCV | 1.341 (1.028 to 1.748) | 0.030 |
| Others | 1.388 (1.124 to 1.714) | 0.002 |
| **Recipient HCV status: positive** | 0.844 (0.685 to 1.040) | 0.112 |
| **Recipient medical condition at TX** |  |  |
| Not Hospitalized | Ref | - |
| Hospitalized, but not in ICU | 0.959 (0.773 to 1.189) | 0.703 |
| In ICU | 1.671 (1.292 to 2.160) | <0.001 |
| **Recipient functional status at TX**  **(per 10 percentage points)**  **(10% - Moribund, 100% - Normal)** | 0.890 (0.854 to 0.928) | <0.001 |
| **Pre-transplant dialysis: yes** | 1.555 (1.269 to 1.905) | <0.001 |
| **Recipient diabetes** |  |  |
| No | Ref | - |
| Type 1 | 1.518 (0.950 to 2.426) | 0.081 |
| Type 2 | 1.093 (0.941 to 1.268) | 0.245 |
| **Log2-Days on liver waiting list** | 1.082 (1.052 to 1.114) | <0.001 |
| **RCS: Donor ALT*** | RCS terms | 0.197 |
| **RCS: Donor creatinine*** | RCS terms | 0.586 |
| **RCS: Year of liver transplantation*** | RCS terms | <0.001 |
| **RCS: Cold ischemic time*** | RCS terms | <0.001 |
| **RCS: Donor admission-to-retrieval time*** | RCS terms | 0.289 |


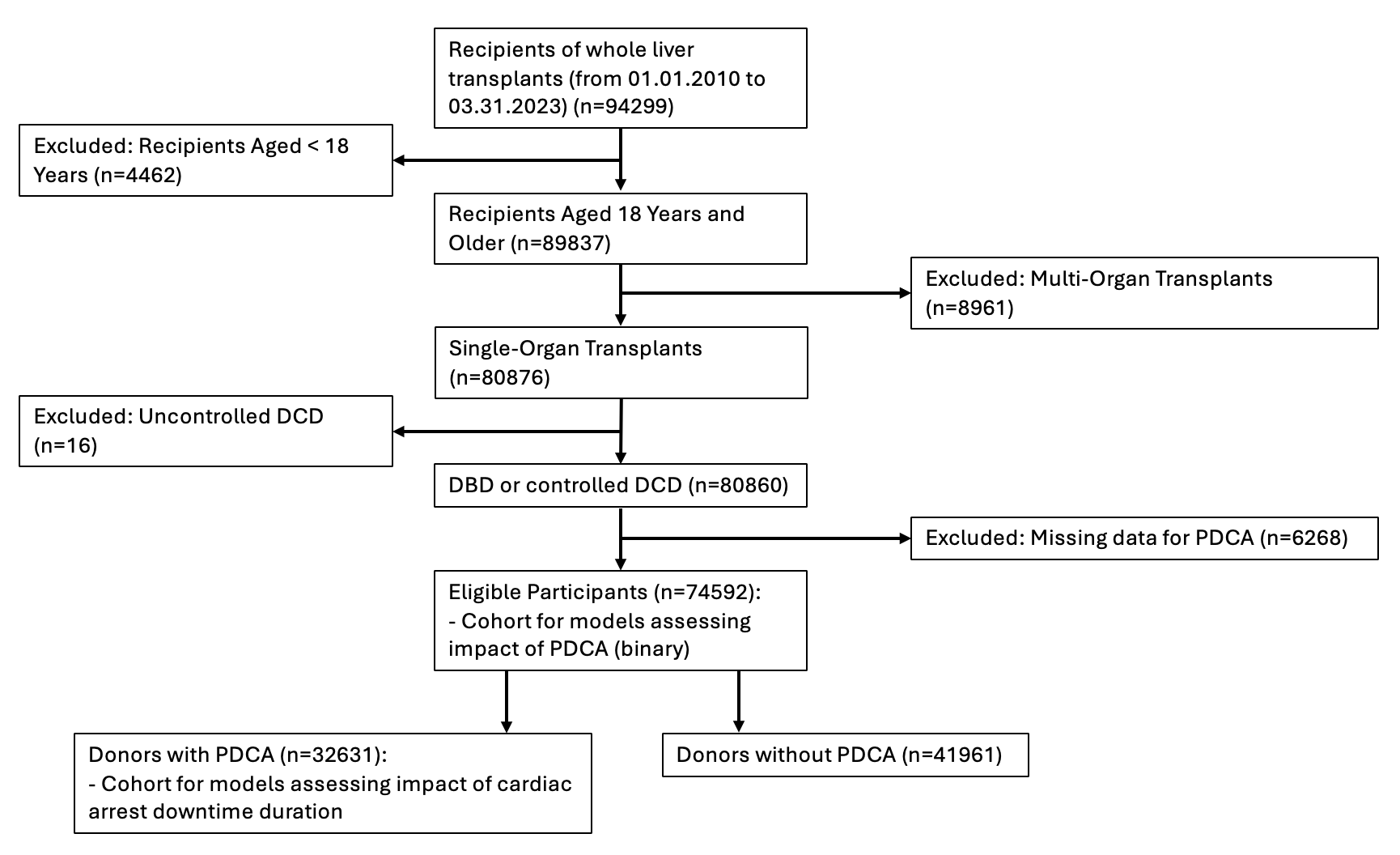


Supplementary Figure 1: Flowchart.

Supplementary Figure 2: Violin plot for donor peak ALT values stratified by PDCA occurrence

Supplementary Figure 3: Associations between (A) donor ALT levels, (B) donor creatinine, (C) cold ischemic time, (D) year of transplant and (E) Donor hospital length of stay plotted against 1-year graft survival for the full cohort utilizing restricted cubic splines with 4 knots. These RCS models are derived from the model presented Table 2.

Supplementary Figure 4: Associations between (A) donor ALT levels, (B) donor creatinine, (C) cold ischemic time, (D) year of transplant and (E) donor hospital length of stay plotted against 1-year patient survival for the full cohort utilizing restricted cubic splines with 4 knots. These RCS models are derived from the model presented in Supplementary Table 2.

Supplementary Figure 5: Associations between (A) donor ALT levels, (B) donor creatinine, (C) cold ischemic time, (D) year of transplant and (E) donor hospital length of stay plotted against length of stay for the full cohort utilizing restricted cubic splines with 4 knots. These RCS models are derived from the model presented in Supplementary Table 3.

##

Supplementary Figure 6: Associations between (A) Donor ALT levels, (B) Donor creatinine, (C) Cold ischemic time, (D) Year of transplant and (E) Donor hospital length of stay plotted against graft loss in 30 days for the full cohort utilizing restricted cubic splines with 4 knots. These RCS models are derived from the model presented in Supplementary Table 4.

Supplementary Figure 7: Associations between (A) donor ALT levels, (B) donor creatinine, (C) cold ischemic time, (D) year of transplant and (E) donor hospital length of stay plotted against 1-year graft survival for the PDCA cohort utilizing restricted cubic splines with 4 knots. These RCS models are derived from the model presented in Table 3.

Supplementary Figure 8: Associations between (A) donor ALT levels, (B) donor creatinine, (C) cold ischemic time, (D) year of transplant and (E) donor hospital length of stay plotted against 1-year patient survival for the PDCA cohort utilizing restricted cubic splines with 4 knots. These RCS models are derived from the model presented in Supplementary Table 6.


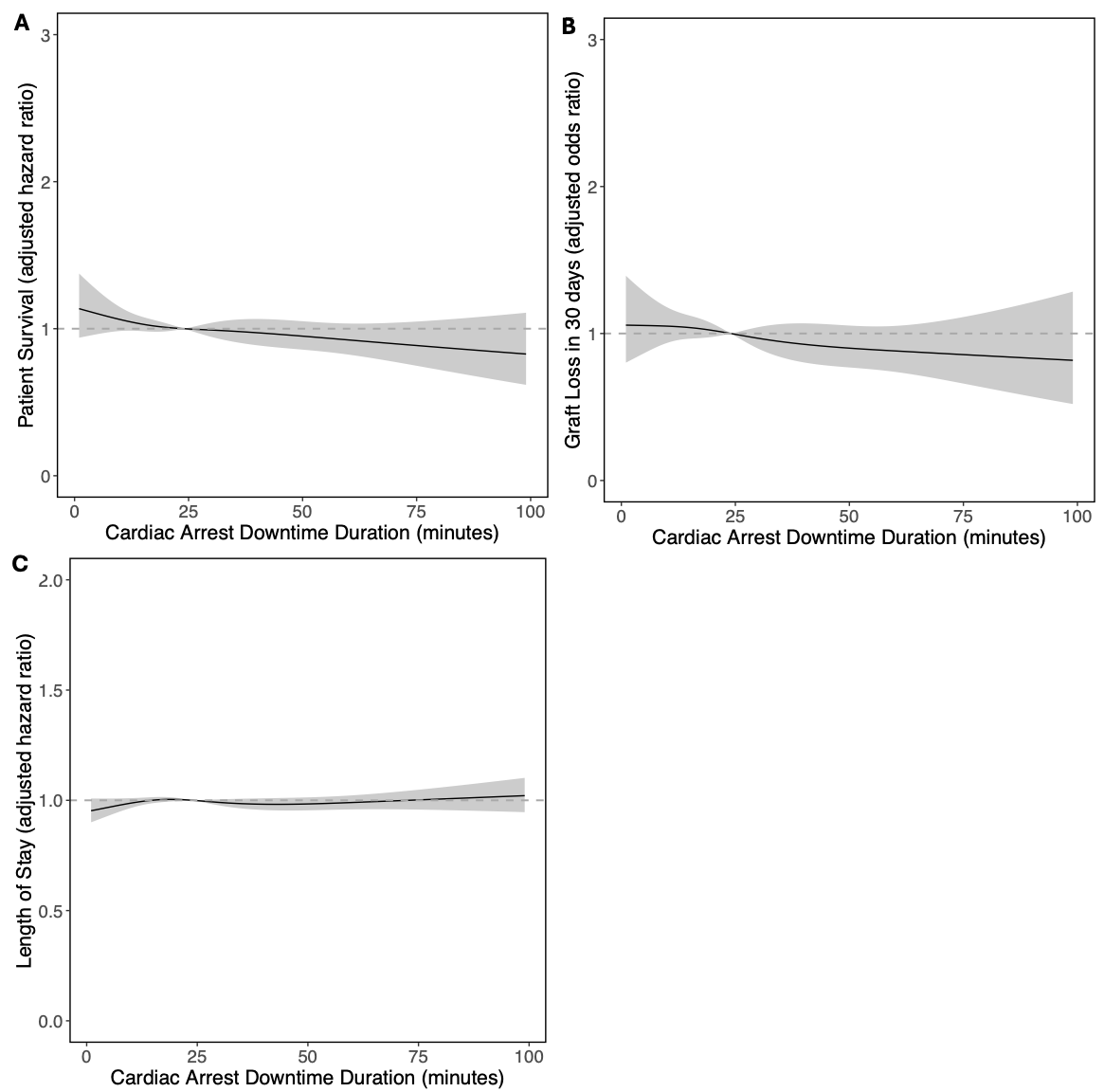


Supplementary Figure 9: Associations between (A) patient survival, (B) length of stay and (C) graft loss in 30 days plotted against PDCA downtime duration utilizing restricted cubic splines with 4 knots.

Supplementary Figure 10: Associations between (A) donor ALT levels, (B) donor creatinine, (C) cold ischemic time, (D) year of transplant and (E) donor hospital length of stay plotted against length of stay for the PDCA cohort utilizing restricted cubic splines with 4 knots. These RCS models are derived from the model presented in Supplementary Table 7.

Supplementary Figure 11: Associations between (A) donor ALT levels, (B) donor creatinine, (C) cold ischemic time, (D) year of transplant and (E) donor hospital length of stay plotted against graft loss in 30 days for the PDCA cohort utilizing restricted cubic splines with 4 knots. These RCS models are derived from the model presented in Supplementary Table 8.
